# Immunosuppressant Treatment in Rheumatic Musculoskeletal Diseases Does Not Inhibit Elicitation of Humoral Response to SARS-CoV-2 Infection and Preserves Effector Immune Cell Populations

**DOI:** 10.3389/fimmu.2022.873195

**Published:** 2022-06-10

**Authors:** Andrea Favalli, Ennio Giulio Favalli, Andrea Gobbini, Elena Zagato, Mauro Bombaci, Gabriella Maioli, Elisa Pesce, Lorena Donnici, Paola Gruarin, Martina Biggioggero, Serena Curti, Lara Manganaro, Edoardo Marchisio, Valeria Bevilacqua, Martina Martinovic, Tanya Fabbris, Maria Lucia Sarnicola, Mariacristina Crosti, Laura Marongiu, Francesca Granucci, Samuele Notarbartolo, Alessandra Bandera, Andrea Gori, Raffaele De Francesco, Sergio Abrignani, Roberto Caporali, Renata Grifantini

**Affiliations:** ^1^ Istituto Nazionale Genetica Molecolare, Padiglione Romeo ed Enrica Invernizzi, Milan, Italy; ^2^ Ph.D. Program in Translational and Molecular Medicine, Dottorato in Medicina Molecolare e Traslazionale (DIMET), University of Milan-Bicocca, Monza, Italy; ^3^ Division of Clinical Rheumatology, Aziende Socio Sanitarie Territoriali (ASST) Gaetano Pini-Centro Traumatologico Ortopedico (CTO) Institute, Milan, Italy; ^4^ Department of Biotechnology and Biosciences, University of Milano-Bicocca, Milan, Italy; ^5^ Department of Clinical Sciences and Community Health, University of Milan, Milan, Italy; ^6^ Department of Pharmacological and Biomolecular Sciences (DiSFeB), University of Milan, Milan, Italy; ^7^ Dia.Pro, Diagnostic Bioprobes srl, Milan, Italy; ^8^ Infectious Diseases Unit, Foundation Istituto Di Ricovero e Cura a Carattere Scientifico (IRCCS) Ca’ Granda Ospedale MaggiorePoliclinico, Milan, Italy; ^9^ Centre for Multidisciplinary Research in Health Science (MACH), University of Milan, Milan, Italy; ^10^ Department of Pharmacological and Biomolecular Sciences, University of Milan, Milan, Italy; ^11^ Department of Clinical Sciences and Community Health, Research Center for Adult and Pediatric Rheumatic Diseases, University of Milan, Milan, Italy

**Keywords:** COVID-19, DMARD, immune responses, rheumatic musculoskeletal diseases, inflammatory arthritis

## Abstract

COVID-19 has proven to be particularly serious and life-threatening for patients presenting with pre-existing pathologies. Patients affected by rheumatic musculoskeletal disease (RMD) are likely to have impaired immune responses against SARS-CoV-2 infection due to their compromised immune system and the prolonged use of disease-modifying anti-rheumatic drugs (DMARDs), which include conventional synthetic (cs) DMARDs or biologic and targeted synthetic (b/ts) DMARDs. To provide an integrated analysis of the immune response following SARS-CoV-2 infection in RMD patients treated with different classes of DMARDs we carried out an immunological analysis of the antibody responses toward SARS-CoV-2 nucleocapsid and RBD proteins and an extensive immunophenotypic analysis of the major immune cell populations. We showed that RMD individuals under most DMARD treatments mount a sustained antibody response to the virus, with neutralizing activity. In addition, they displayed a sizable percentage of effector T and B lymphocytes. Among b-DMARDs, we found that anti-TNFα treatments are more favorable drugs to elicit humoral and cellular immune responses as compared to CTLA4-Ig and anti-IL6R inhibitors. This study provides a whole picture of the humoral and cellular immune responses in RMD patients by reassuring the use of DMARD treatments during COVID-19. The study points to TNF-α inhibitors as those DMARDs permitting elicitation of functional antibodies to SARS-CoV-2 and adaptive effector populations available to counteract possible re-infections.

## Introduction

The COVID-19 pandemic represents a challenge for health systems worldwide. SARS-CoV-2 infection has proven to be particularly dangerous, in terms of both morbidity and mortality, for patients presenting with pre-existing pathologies (1, 2). Patients at risk include those affected by rheumatic musculoskeletal disease (RMD), in which the ability to mount a productive immune response to SARS-CoV-2 infection could be challenged by two intrinsic aspects. First, RMDs are immune-mediated diseases, with a compromised immune system that may cause an altered inflammation status and increased complications upon infection (3, 4). Second, RMD patients are treated with immunosuppressive agents that can blunt immune responses and make them more susceptible to infections, causing a more severe course of infection compared to the general population (3, 5). These patients are generally treated with disease-modifying anti-rheumatic drugs (DMARDs) aimed at slowing disease progression. DMARDs comprise different drugs and mode of actions, but can be categorized as conventional synthetic (cs) DMARDs or biologic and targeted synthetic (b/ts) DMARDs. Some DMARD treatments, such as hydroxychloroquine, anti-TNFα, and IL-6 inhibitors, have been employed during the COVID-19 pandemic to reduce the systemic inflammation associated with severe disease and are being studied for the prevention and/or treatment of COVID-19 and its complications (6–9).

To date, a limited number of studies investigated the risk of infection or COVID-19 severity in RMD patients, associating seroprevalence of anti-SARS-CoV-2 antibodies. These studies are reassuring about the low impact of RMDs and immunomodulatory therapies on the risk and clinical course of COVID-19 (10–12).

However, at present, the insight into the immune response to SARS-CoV-2 in RMD patients has been limited to seroprevalence analyses, while a global picture of the immune response relevant for protective immunity against SARS-CoV-2 reinfection is still missing. In this frame, our work aimed at describing the immune response following SARS-CoV-2 exposure raised in RMD patients treated with different classes of DMARDs. We show that RMD individuals under most DMARD treatments mount a sustained antibody response to the virus and effector T and B lymphocytes. In particular, these patients are able to elicit neutralizing antibodies titers comparable to non-RMD COVID-19 patients, potentially able to counteract SARS-CoV-2 infection. Among b-DMARDs, our study highlights anti-TNFα treatments as more propitious drugs to mount a humoral and cellular immune response as compared to CTLA4-Ig and anti-IL6R antibodies.

## Materials and Methods

### Patient Recruitment

The study involved two patient recruitments, the first in May–June 2020 (T1) and the second in September–October 2020 (T2). Recruitments occurred at the ASST Gaetano Pini-CTO Institute in Milan (Italy) and IRCCS Ca’ Granda Ospedale Maggiore Policlinico Foundation, and were under ethical approval by the Ethics Committee Milano Area 2 (MAINSTREAM protocol: approval number 407; END-COVID: approval number 331). All patients signed informed consent.

The study population at T1 was recruited 78.8 days (median, range 24–111) after the presentation of COVID-19 symptoms, including 358 RMD patients with a diagnosis of rheumatoid arthritis (RA, *N* = 200, 56%) or other diseases [ankylosing spondylitis, spondyloarthritis (SpA), *N* = 158, 44%] receiving treatments with DMARD (**Supplementary Table 1**). These comprised conventional-synthetic (cs)-DMARDs (metotrexate, leflunomide, sulfasalazine, hydroxychloroquine, cyclosporins, and mesalazine), biological (b)-DMARDs (anti-TNF-α mAbs: infliximab, etanercept, adalimumab, certolizumab, and golimumab; anti-IL-6R mAbs: tocilizumab and sarilumab; decoy CTLA-4: abatacept; anti-IL23 mAb: ustekinumab; anti-IL17A mAbs: secukinumab and ixekizumab; anti-CD20 mAb: rituximab; IL1-RA/anti-IL1β: anakinra and canakinumab), targeted-synthetic (ts)-DMARDs (JAK1/2 inhibitor: baricitinib; JAK1/3 inhibitor: tofacitinib; PDE4 inhibitor: apremilast), either alone or in combination. Approximately one-third of the patients were under concurrent treatment with glucocorticoids. Demographic and clinical data of the patients are reported in **Supplementary Table 1**. SpA patients were gender-balanced (F = 75, M = 83), while the RA patient’s group had a predominance of women (F = 155; M = 45) in all treatment classes, in agreement with the expected gender incidence of this disease.

The 277 patients under b-DMARD treatment were equally distributed between RA (*N* = 141) and SpA (*N* = 136). Among the 22 ts-DMARD-treated patients, the majority was affected by RA (*N* = 16). Within the cs-DMARD recruited cohort (*N* = 59), RA was more represented (*N* = 43) as compared to SpA pathologies (*N* = 16). All patients had an established disease of long duration (median, 15 years). About one-third of the patients had at least one comorbidity, including hypertension (24.8%), obesity (8.9%), and cardiovascular disease (3.1%).

Due to the limited availability of the PCR test during the recruitment period (May–June 2020) and the limitations imposed by the strict lockdown and quarantine rules during the first wave of the pandemic, a total of 25 patients (37.87%) who were found to be seropositive to SARS-CoV-2 proteins underwent validation testing after an average period of 123.2 days from symptom onset. Characterization of patients according to COVID-19-related symptoms is provided in **Supplementary Table 2**.

A second recruitment 3–4 months after (T2) allowed whole blood sampling from 36 individuals, 28 of whom were also recruited at T1 (24 treated with b/ts-DMARDs and 4 with cs-DMARDs, **Supplementary Table 1**). Such recruitment included consecutive patients referred to outpatient clinic. Median time of blood drawn after COVID-19 symptoms presentation was 189 days (range, 138–234) in T2. These were predominantly affected by RA (*N* = 23). Also in the T2 cohort, the RA group was predominantly composed of female patients (82.6%), while the SpA group was gender-balanced.

Finally, 13 individuals who had a diagnosis of COVID-19 from April to August 2020 were included as non-RMD controls.

### Cloning of SARS-CoV-2 RBD and N proteins

Human codon-optimized nucleotide sequences encoding SARS-CoV-2 receptor binding domain (RBD; residues 318–541) and the full-length nucleocapsid protein (N) were purchased from Genscript (SARS-CoV-2 sequence isolate: GenBank MN908947-Wuhan-Hu-1) and subcloned into the mammalian expression vector pcDNA 3.4. Recombinant constructs included an N-terminal signal peptide optimized for protein expression and secretion, and a C-terminal octa-histidine tag for purification. For recombinant protein production, 7.5 × 10^7^ Expi293 cells (Expi293™ Expression System, Thermo Fisher Scientific), seeded in 25 ml medium, were transiently transfected with ExpiFectamine293 containing 1 μg/ml of the resulting plasmids, according to the manufacturer’s instructions. Seventy-two hours after transfection, recombinant proteins were purified using Ni_2_^+^-NTA affinity chromatography (ÄKTA™ Pure, Cytiva). Briefly, supernatants were clarified by centrifugation and buffer exchanged with binding buffer (20 mM Tris, 10 mM NaCl, and 10 mM imidazole, pH 8) using a HiPrep™ 26/10 desalting column. Individual supernatants were then loaded onto a nickel-chelating resin pre-equilibrated with binding buffer. The resin was washed with wash buffer containing 30 mM imidazole and eluted with the same buffer containing 300 mM imidazole at pH 8. Peak fractions were analyzed by SDS-PAGE and those corresponding to soluble proteins were pooled, buffer exchanged using a HiPrep™ 26/10 desalting column running in 1× PBS and stored at 4°C.

### ELISA Assays

ELISA assays were conducted by Diapro SrL using a proprietary and CE IVD Certified commercial diagnostic kit (COVID-19 IgG/IgM ELISA), which had been validated by the manufacturer using international standards and clinically validated in different countries, in compliance with commercialization requirements for IVD available in 2020.

In essence, 96-well flat-bottom Immulon 2HB plates (Thermo Fisher Scientific) were coated (Freedom-EVO Liquid Handling system, Tecan) with 100 μl of purified recombinant protein solution (2.5 μg/ml in PBS) and placed at 4°C overnight. Plates were washed once (Hydrospeed™ -Tecan) with wash buffer (PBS + 0.05% Tween 20) and 150 μl of ELISA blocking buffer (PBS, 5% BSA - Sigma-Aldrich) was added to the well. Plates were incubated for 1 h at 37°C. After washing (×3) with Hydrospeed™ (Tecan), 100 μl of sample sera were added to each well. Sera were tested in eight serial 2-fold dilutions (in PBS + 0.5% Tween20 with 1% BSA-dilution buffer) starting at 1:100, and incubated for 1 h at 37°C. Plates were washed 4 times with wash buffer and probed with 100 μl of HRP conjugated α-human IgG, IgM, and IgA secondary antibodies (Sigma-Aldrich) at a dilution of 1:1,000 (in dilution buffer) for 40 min at room temperature. Plates were washed 3 times with Hydrospeed™ (Tecan), and 100 μl of TMB substrate (Thermo Fisher Scientific) was added to the wells and plates were incubated for 10–15 min in the dark at room temperature. The reaction was stopped by adding 100 μl of 1 M H_2_SO_4_, and the absorbance was measured at λ = 450 nm by Infinite F200 PRO instrument (Tecan). Absorbance values of the Negative Control (NC) plus 3 standard deviation values were defined as cutoff. Results were interpreted as a ratio of the sample OD450 value (S) and the cutoff value (Co), or S/Co. Ratio values higher than 1.5 were considered as positive, according to the manufacturer’s instruction. The cutoff was set to S/Co = 1.5 to minimize false-positive/-negative results. Such low ratio also avoided exclusion of patients who mounted very low response due to the immune-suppressant treatments.

### Generation of hACE2-Expressing Cell Line

A cell line stably expressing hACE2 receptor (HEK293TN-hACE2) was generated by lentiviral transduction of HEK293TN cells (obtained from System Bioscience). Lentiviral vectors were produced following a standard procedure based on calcium phosphate cotransfection with 3rd-generation helper and transfer plasmids. The following helper vectors were gifts from Didier Trono: pMD2.G/VSV-G (Addgene #12259), pRSV-Rev (Addgene #12253), and pMDLg/pRRE (Addgene #12251). The transfer vector pLENTI_hACE2_HygR was obtained by cloning of hACE2 coding sequence from pcDNA3.1-hACE2 (a gift from Fang Li, Addgene #145033) into pLenti-CMV-GFP-Hygro (a gift from Eric Campeau & Paul Kaufman, Addgene #17446). hACE2 was amplified by PCR and inserted under the CMV promoter of the pLenti-CMV-GFP-Hygro after GFP excision with *Xba*I and *Sal*I digestion. pLENTI_hACE2_HygR is now available to the scientific community through Addgene (Addgene #155296). The hACE2 lentiviral vector obtained was used to transduce HEK293TN. Forty-eight hours after transduction, cells were selected through hygromycin exposure (250 μg/ml). Ectopic expression of hACE2 was confirmed by flow cytometry using Anti-hACE2 primary antibody (AF933, R&D system, 0.75 μg/200,000 cells) and rabbit anti-goat IgG (Alexa Fluor 647) secondary antibody (1:200 in PBS + 2% FBS). The expression of hACE2 was observed in more than 90% of the cells and found to be stable after several passages. HEK293TN-hACE2 cells were maintained in DMEM, supplemented with 10% FBS, 1% glutamine, 1% penicillin/streptomycin, and 250 μg/ml Hygromycin (GIBCO).

### Inhibition of Binding Assay

Purified recombinant SARS-CoV-2 RBD protein was labeled with Alexa Fluor 647 (Alexa Fluor 647 Microscale protein Labeling Kit #A30009 Invitrogen) according to the manufacturer’s instructions. The binding of labeled RBD on HUH7.5 cells (which endogenously express high levels of hACE2) was confirmed at different protein concentrations and maximum binding was obtained using RBD at 10 μg/ml.

To assess the content of inhibiting antibodies in sera, 10 μl of SARS-CoV-2 RBD-Alexa Fluor 647 at 10 μg/ml in PBS-1% FCS were mixed with 10 μl of various dilutions of sera in conical 96-well plates for 30 min at 37°C. After incubation, 3 × 10^4^ HUH7.5 cells suspended in 5 μl of PBS-1% FCS were added and incubated for an additional 1 h at 37°C. Unbound protein and antibodies were removed with two washes in PBS and cell-bound Alexa Fluor 647 fluorescence was analyzed with a FACSCanto-II flow cytometer (BD Biosciences). Data were analyzed using the FlowJo data analysis software package v.10 (LLC). The % of binding inhibition was calculated as follows: IOB (%) = 1 − (Sample MFI value − background MFI value)/(Negative Control MFI value − background MFI value).

### SARS-CoV-2 Pseudovirus Generation and Titration

To generate SARS-CoV-2 pseudoviral particles, 5 × 10^6^ HEK-293TN cells were plated in a 15-cm dish with complete DMEM medium. The following day, 32 µg of reporter plasmid pLenti CMV-GFP-TAV2A-LUC Hygro, 12.5 µg of pMDLg/pRRE (Addgene #12251), 6.25 µg of pRSV-Rev (Addgene #12253), and 9 μg of pcDNA3.1_ spike_del19 were co-transfected following a standard procedure based on calcium phosphate transfection. pLenti CMV-GFP-TAV2A-LUC Hygro was generated from pLenti CMV GFP Hygro (Addgene #17446) by the addition of T2A-Luciferase by PCR cloning. pcDNA3.1_spike_del19 was generated by the deletion of the last 19 aa of spike starting from pcDNA3.1-SARS2-Spike [a gift from Fang Li, Addgene plasmid # 145032 (13)] and is now available through Addgene (Addgene #155297). After 12 h, the medium was changed with 16 ml of complete ISCOVE for each dish. Thirty hours after transfection, the supernatant was collected, clarified by filtration with a 0.45-μm pore-size filter (VWR 514-0331), and concentrated 400× by centrifugation for 2 h at 20,000 rpm using SW32Ti. Pseudoviral particles were aliquoted and stored at −80°C.

Pseudovirus preparation was titrated in HEK-293TN hACE2. Cells were seeded at 2.5 × 10^4^ cells/well in 24-well plates. After 24 h, cells were transduced with 5 serial threefold dilution of pseudovirus, starting from 5 µl of concentrated preparation. A negative control of non-transduced cells was included. After 48 h, cells were collected, washed with PBS, and fixed in PFA 1%. Flow cytometry analysis was performed to determine the percentage of GFP-positive HEK293TN hACE2 that correlates to the number of pseudoviral particles contained in the preparation. Pseudovirus titer (PV/ml) is calculated among the linear range of the obtained transduction curve (4%–20% of cells infected) and was used to define the MOI in the neutralization assay.

### Neutralization of Infection With Pseudoviral Particles

For neutralization assay, HEK293TN-hACE2 was plated at 10^4^ cells/well in white 96-well plates (100 µl/well of complete DMEM medium). After 24 h, cells were transduced with 0.1 MOI of SARS-CoV-2 pseudovirus previously incubated with serial threefold dilution of serum in order to obtain a 7-point dose–response curve. Briefly, 5 µl of each dilution was added to 45 µl of DMEM medium containing the pseudovirus and incubated for 1 h at 37°C. Fifty microliters of the serum/pseudovirus mixture was then added to each well and plates were incubated for 24 h at 37°C. Each point was assayed in triplicate. After 24 h of incubation, cell infection was measured by luciferase assay using the Bright-Glo™ Luciferase System (Promega). An Infinite F200 plate reader (Tecan) was used to read luminescence. Obtained RLUs were normalized to controls and dose–response curves were generated by a nonlinear regression curve fitted with GraphPad Prism to calculate Neutralization Dose 50 (ND50).

### Immunophenotyping by High-Dimensional Flow Cytometry

Peripheral blood mononuclear cells (PBMCs) from RMD patients at T2 or convalescents were isolated by density-gradient centrifugation using Ficoll-Paque PLUS (GE Healthcare). For each staining, 100,000 cells were washed in PBS and incubated with Fixable Viability Stain 780 (BD Horizon, cat n° 565388) diluted 1:2,000 in PBS at room temperature in the dark. After 15 min, cells were washed in PBS. For surface marker detection, cells were incubated in Brilliant Stain Buffer (BD Horizon, cat n° 566349) diluted 1:2 in PBS and supplemented with antibodies for 30 min at room temperature in the dark. Cells were then washed in PBS and fixed 15 min at 4°C using the eBioscience FOXP3 staining kit according to the manufacturer’s protocol (eBioscience, cat n° 00-5523). To detect intracellular factors (EOMES, FOXP3; GZMK; GZMB), a further incubation in Permeabilization Reagent (eBioscience, cat n° 00-833) supplemented with antibodies was performed for 30 min at 4°C.

For intracellular cytokine staining, at least 100,000 cells were stimulated for 2 h at 37°C in a 5% CO_2_ atmosphere with 100 ng/ml phorbol myristate acetate (PMA) and 2 μg/ml ionomycin (Sigma-Aldrich) in culture medium (RPMI 1640 supplemented with 10% FBS, 1% each of L-glutamine, sodium pyruvate, non-essential amino acids, and Pen/Strep). A total of 100,000 cells were left unstimulated as negative control. Samples were cultured with Brefeldin A (BFA) for an additional 2 h and then washed with PBS. Staining procedure was performed as described above. Samples were acquired on a BD FACSymphony A5 cytometer (BD Biosciences) equipped with 5 lasers (UV, 350 nm; violet, 405 nm; blue, 488; yellow/green, 561 nm; red, 640 nm). Markers used for immunophenotype staining of immune cell subsets are listed in **Supplementary Table 3**. Antibodies used for high-dimensional flow cytometry analyses are listed in **Supplementary Table 4**. The gating strategy used to identify the different populations is illustrated in **Supplementary Figure 1**.

### RT-qPCR

CD14^+^ cells were isolated from PBMCs using CD14 MicroBeads (cat #130-050-201). CD14 cells were cultured in RPMI-1640 (Euroclone) supplemented with 10% heat-inactivated fetal bovine serum (Gibco), 100 U/ml penicillin (Euroclone), 0.1 mg/ml streptomycin (Euroclone), MEM non-essential amino acids (GIBCO), 2 mM L-glutamine (Euroclone), 10 mM HEPES buffer solution (GIBCO), and 1 mM sodium pyruvate (GIBCO), and stimulated with poly (I:C) at 500 ng/ml (InvivoGen) or left untreated as a control. Twenty-four hours post stimulation, cellular RNA was isolated using PureLinkTM RNA Mini Kit (Invitrogen). RNA was treated with PureLinkTM DNase (Invitrogen). cDNA was generated using the SuperScript^®^VILOTM cDNA synthesis kit (Thermo Fisher Scientific). Gene expression was measured by quantitative real-time PCR using the SensiFAST sybr LO-ROX kit (Bioline) according to the manufacturer’s procedures in a QuantStudio 5 Real-Time PCR System (Applied Biosystem). RSP11 was used as a housekeeping gene to normalize the data. Cytokine gene expression was determined using QuantiTect primer assays from Qiagen.

### Statistical Analysis

All the statistical analysis and plots were done with R language (v. 4.2.1) and GraphPad Prism (v. 9.2). Statistical significance of all pairwise comparisons was assessed by two-tailed nonparametric tests; Mann–Whitney for unpaired data and Wilcoxon signed rank tests for paired data. Kruskal–Wallis tests were used to compare unpaired samples between multiple study groups. Multiple comparisons were corrected using Dunn’s test. Correlation analysis was computed as non-parametric Spearman correlation, while Fisher’s exact test was applied for categorical variables’ analysis. Only significant comparisons were reported above the plots (*p < 0.05, **p < 0.01, ***p < 0.001, ****p < 0.0001). Specific details of other statistical analysis are found in the figure legends.

## Results

### COVID-19 Symptomatic RMD Patients Showed a Seropositivity Rate to SARS-CoV-2 Infection, Independently on b/ts-DMARD or cs-DMARD Treatments

During the first COVID-19 pandemic wave in spring 2020 (T1), we collected 358 serum samples from RMD patients with a diagnosis of rheumatoid arthritis (RA, *N* = 200) or other diseases (ankylosing spondylitis, spondyloarthritis; hereafter SpA, *N* = 158) receiving treatments with DMARDs (**Supplementary Table 1**), comprising cs-DMARDs (mainly methotrexate) (*N* = 59) and b/ts-DMARD (*N* = 299), either alone or in combination (**Supplementary Table 1**). Among b-DMARDs, anti-TNF-α, anti−IL-6R, and CTLA4-Ig were the most common (**Supplementary Table 1**). Approximately one-third of the patients were under concurrent treatment with low-dose glucocorticoids (mean dose 4 mg daily, prednisone equivalent). SpA patients were gender-balanced (F = 75, 47.5%), while the RA patient’s group had a predominance of women (F = 155, 77.5%), in agreement with the expected gender incidence of this disease (**Supplementary Table 1**).

Patients (358) were initially considered as “suspected COVID-19” based on occurrence of COVID-19-related symptoms (**Supplementary Table 5**) (14). Patients reporting at least 1 major and 2 minor symptoms, or at least 2 major symptoms (21.5%) were classified as “COVID-19 symptomatic” while those reporting only minor symptoms (78.5%) were considered as “COVID-19 asymptomatic” (**Supplementary Table 6**).

Confirmation of COVID-19 was assessed by measuring the serological response (IgM, IgG, and IgA) to SARS-CoV-2 receptor binding domain (RBD) and/or nucleocapsid (N) proteins through ELISA. Seropositive patients were scored based on the presence of at least one Ig class (IgM/IgG/IgA) with signal-to-control ratio (S/Co) values to N and/or RBD >1.5 (this score was set to also detect a low-level immunological response to COVID-19 in this immunosuppressed population). According to this criterion, 18.4% of patients were seropositive to SARS-CoV-2, and were therefore considered as confirmed COVID-19 (**Table 1**). Seroprevalence was independent on demographic and annotated lifestyle variables (e.g., age, gender, smoking) (19).

**Table 1:** Anti-RBD seroprevalence of RMD patients and associated clinical features.

	Serology positive *N* (%)	IgM*N* (%)	IgG*N* (%)	IgA*N* (%)	IgM+IgG+IgA*N* (%)	Total number
COVID symptomatic	25 (32.5)	19 (24.7)	17 (22.1)	17 (22.1)	12 (15.6)	77
COVID asymptomatic	41 (14.6) ***	23 (8.2) ****	13 (4.6) ****	26 (9.3) **	8 (2.8) ***	281
RA	36 (15)	23 (11.5)	20 (10)	27 (13.5)	14 (7)	200
SpA	30 (15)	19 (12)	10 (6.3)	16 (10.1)	6 (3.8)	158
b/ts-DMARD	55 (18.4)	35 (11.7)	24 (8)	36 (12)	17 (5.7)	299
cs-DMARD	11 (18.6)	7 (11.9)	6 (10.2)	7 (11.9)	3 (5.1)	59
b/ts ONLY	42 (22.5)	27 (14.4)	19 (10.2)	27 (14.4)	14 (7.5)	187
COMBO ONLY	26 (23.2)	8 (7.1)	8 (7.1)	9 (8)	3 (2.7)	112
a-TNFa	37 (21.4)	25 (14.5)	16 (9.1)	21 (12.1)	10 (5.8)	173
a-IL-6R	8 (22.9)	5 (14.3)	6 (17.1)	8 (22.9)	5 (14.3)	35
CTLA4-Ig	5 (11.9)	3 (7.1)	1 (2.4)	3 (7.1)	1 (2.4)	42
w/wo PDN				
b/ts-DMARD	36 (7.2)	22 (10.6)	14 (6.8)	22 (10.6)	9 (4.3)	207
b/ts-DMARD + PDN	19 (19.6)	13 (14.1)	10 (10.9)	14 (14.1)	8 (8.7)	92
a-TNFa	29 (16)	18 (13)	10 (7.2)	16 (11.6)	6 (3.5)	138
a-TNFa + PDN	8 (22.9)	7 (17)	6 (17.1)	5 (14.3)	4 (11.43)	35
a-IL-6R	4 (22.2)	2 (11.1)	3 (16.7)	4 (22.2)	2 (11.1)	18
a-IL6R + PDN	4 (23.5)	3 (17.6)	3 (17.6)	4 (23.5)	3 (17.6)	17
CTLA4-Ig	2 (9.5)	0 (0)	0 (0)	2 (9.5)	0 (0)	21
CTLA4-Ig + PDN	3 (14.3)	3 (14.3)	1 (4.8)	1 (4.8)	1 (4.8)	21
cs	10 (21.7)	7 (15.2)	6 (13)	6 (13)	3 (6.5)	46
cs + PDN	1 (7.7)	0 (0)	0 (0)	1 (7.6)	0 (0)	13
b/ts-DMARD RA	28 (17.8)	17 (10.8)	15 (9.6)	23 (14.6)	12 (7.6)	157
b/ts-DMARD SpA	27 (15)	18 (12.7)	9 (6.3)	13 (9.2)	5 (3.5)	142
a-TNFa RA	11 (18)	7 (11.5)	7 (11.5)	9 (14.8)	5 (8.2)	61
a-TNFa SpA	26 (23.2)	18 (16.1)	9 (8)	12 (10.7)	5 (4.5)	112
a-IL-6R RA	8 (23.5)	5 (14.7)	6 (17.6)	8 (23.5)	5 (14.7)	34
a-IL6R SpA	0 (0)	0 (0)	0 (0)	0 (0)	0 (0)	1
CTLA4-Ig RA	5 (12.8)	3 (7.7)	1 (2.6)	3 (7.7)	1 (2.6)	39
CTLA4-Ig SpA	0 (0)	0 (0)	0 (0)	0 (0)	0 (0)	3
cs RA	8 (18.6)	6 (14)	5 (11.6)	4 (9.3)	2 (4.7)	43
cs SpA	3 (18.8)	1 (7.7)	1 (7.7)	3 (23.1)	1 (7.7)	16

Statistical analysis among groups was determined with Chi-squared and Fisher’s exact test. Differences in the seropositive rates between symptomatic and asymptomatic patients are marked by parentheses (p-value: **p < 0.01; ***p < 0.001; ****p < 0.0001).

**Figure 1:**
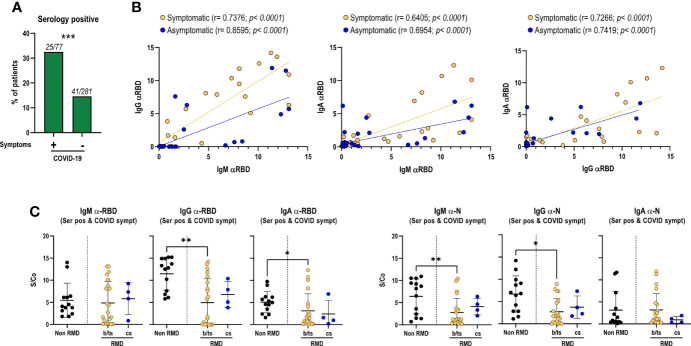
Magnitude of the anti-RBD antibody response in RMD patients, associated with the occurrence of COVID-19 symptoms and treatment categories, as compared to non-RMD individuals. Levels of IgM, IgG, and IgA to SARS-CoV-2 N and RBD measured by ELISA in sera of RMD patients treated with b/ts-DMARD and cs-DMARD and non-RMD individuals who recovered from COVID-19. **(A)** Frequency of patients symptomatic (*N* = 77) and asymptomatic (*N* = 281) for COVID-19 that were seropositive to N or RBD proteins. Fisher’s exact test was used as statistical analysis. **(B)** Correlation analysis between anti-RBD IgM/G/A levels in serology-positive COVID-19 symptomatic (*N* = 25) and asymptomatic (*N* = 41) RMD patients. The *p*-value and correlation coefficient *r* were calculated using Pearson correlation. **(C)** Levels of each anti-RBD and anti-N Ig class in serology positive and symptomatic patients’ groups classified by DMARD treatment categories. b/ts-DMARD, *N* = 21; cs-DMARD, *N* = 4; non-RMD, *N* = 13. Graphs represent individual values, means, and SD. Statistical significance was determined using two-tailed Mann–Whitney test. Asterisks denote differences with statistical significance among groups. Statistical significance are reported as: *p < 0.05; **p < 0.01, ***p < 0.001.

COVID-19 symptomatic RMD patients had a higher seropositivity rate (32.5%) than asymptomatic ones (14.6%) (*p*-value = 0.0003) (**Figure 1A**). Seroprevalence did not significantly differ between RA and SpA groups, and was also comparable between patients treated with b/ts-DMARD or cs-DMARDs, either alone or in combination (**Table 1**).

Although treatment with glucocorticoids has been reported to have an impact on the response to SARS-CoV-2 infection (15, 17, 18, 20, 21), we did not detect a relevant impact of prednisone treatment on the seroconversion rate in patients treated with either b/ts-DMARDs or cs-DMARD alone or in combination with prednisone (**Table 1**). However, this modest impact specifically refers to patients undergoing therapy with low prednisone doses. Finally, seroprevalence was not different between RA and SpA patients in the distinct treatment groups (**Table 1**).

Overall, these data show a significant association between seropositivity and COVID-19-related symptomatology. Moreover, the data indicate that b/ts-DMARDs or cs-DMARDs do not differentially affect seropositivity rate in RMD patients.

### All DMARDs Except CTLA4-Ig Allow Induction of a Sustained Serological Response to SARS-CoV-2

Since a high antibody response generally reflects a protective immunity to SARS-CoV-2 infection, we measured the level of the antibody response to SARS-CoV-2 proteins in the 66 RMD seropositive patients. We included a cohort of non-RMD individuals recovered from COVID-19 with moderate symptomatology (*N* = 13 collected in the same period, at >21 days from COVID-19 diagnosis) (**Supplementary Table 1**). This cohort was used as a reference group of individuals who had successfully resolved the SARS-CoV-2 infection in the absence of immune-suppressive treatments.

By analyzing the relative abundance of IgM, IgA, and IgG elicited against RBD, particularly relevant to prevent viral reinfection, we found a positive correlation between IgM and IgG/IgA, and between IgG and IgA, indicating a balanced immunoglobulin seroconversion during infection (**Figure 1B**). Such positive correlation was maintained irrespective of COVID-19 symptomatology, suggesting that symptom occurrence did not significantly impact the production of virus-specific antibodies (**Figure 1B**). Comparable antibody levels against RBD and N proteins were observed between patients treated with b/ts-DMARDs and cs-DMARDs, indicating that these treatment macro-categories did not differentially influence the magnitude of the antibody response to SARS-CoV-2 (**Supplementary Figure 2A**), nor did concomitant treatment with glucocorticoids (**Supplementary Figure 2B**). Overall, RMD patients mounted a lower level of SARS-CoV-2-specific antibodies compared to non-RMD individuals, in line with expectations (**Supplementary Figure 1A**, **Supplementary Figure 2A**). Nevertheless, when focusing on symptomatic RMD patients, the difference was less pronounced (**Figure 1C**).

These data strengthen the evidence that DMARD treatments reduce but do not inhibit the elicitation of the antibody response, without relevant differences between b/ts- or cs-DMARD treatments.

We then hypothesized that the development of a productive antibody response to SARS-CoV-2 infection could vary in different b-DMARD treatments, depending on their mechanisms of action. We focused on anti-TNF-α (Infliximab, Etanercept, Adalimumab, Certolizumab, and Golimumab), anti-IL-6R (Tocilizumab and Sarilumab), and CTLA4-Ig treatments (Abatacept), as they were more represented in our cohort, and compared them to cs-DMARD-treated patients. In line with what was globally observed for b/ts-DMARDs, patients undergoing different b-DMARDs produce a lower antibody response than non-RMD individuals, especially anti-RBD IgG-IgA and anti-N IgG, with CTLA4-Ig-treated patients having the lowest IgG levels (**Supplementary Figure 3A**). This difference appeared to be less marked when restricting the analysis to the serologically positive RMD patients symptomatic for COVID-19 (**Supplementary Figure 3B**).

### RMD Patients Elicit Neutralizing Antibodies

**Figure 2:**
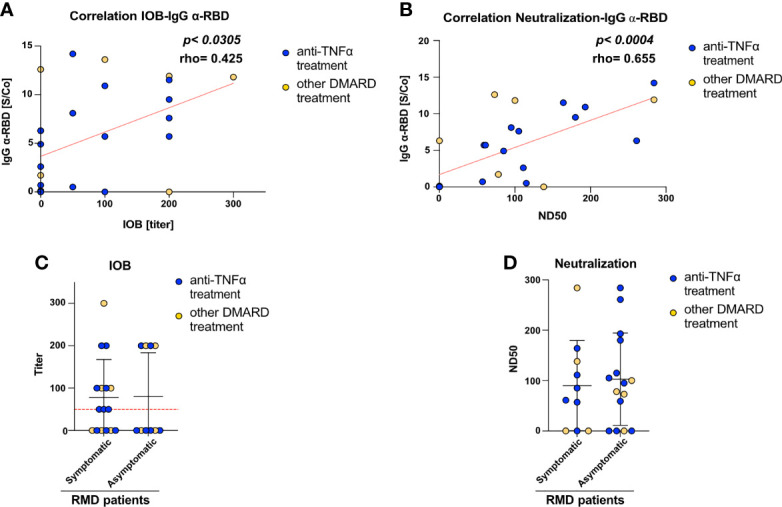
RMD patients elicit anti-SARS-CoV-2 neutralizing antibodies. Correlation between **(A)** IOB and **(B)** ND50 (neutralization dose 50 vs. anti-RBD IgG levels in RMD patients at T1. In the graph, samples from anti-TNFα-treated patients are marked by color code. The *p*-value and correlation coefficient rho were calculated using non-parametric Spearman correlation. **(C)** Inhibition of binding of recombinant RBD protein to HuH7.5 cell line expressing hACE2 by sera of RMD patients stratified by occurrence of COVID-19 symptoms. **(D)** Neutralization of infection with SARS-CoV-2 pseudoparticles of RMD patients’ sera, symptomatic and asymptomatic, were tested for their ability to neutralize pseudotyped viral particle infection. Samples from anti-TNFα-treated patients are marked by color code. *N* = 26. Statistical significance was determined using two-tailed Mann–Whitney test.

To get insights into the functionality and neutralization ability of the virus-specific antibodies detected, we performed both inhibition of binding (IOB) and neutralization of infection with SARS-CoV-2 pseudovirus assays. The IOB assay evaluates the ability of patients’ sera to inhibit the binding of a fluorescently labeled recombinant RBD protein to HUH7.5 cells expressing hACE2 receptor. The neutralization assay, based on an *in vitro* infection of SARS-CoV-2 S protein pseudotyped particles on HEK293TN expressing hACE2 receptor, is a surrogate assay to evaluate the ability of patients’ sera to inhibit infection. Results from both assays indicated that anti-RBD IgG measured at T1 showed neutralization activity; in fact, a positive and statistically significant correlation can be appreciated between anti-RBD IgG and both IOB titer (*p* < 0.0305, **Figure 2A**) and ND50 (*p* < 0.004, **Figure 2B**).

The neutralization assay with viral pseudoparticles showed that a high percentage (72%) of RMD patients elicited neutralizing sera. The IOB assay confirmed that 53.8% of these sera having anti-RBD antibodies were able to inhibit the binding of RBD in the assay (IOB titer ≥ 1:50). Remarkably, both neutralization and IOB titers at T1 were comparable between the symptomatic and asymptomatic groups (**Figures 2C, D**). Highlighting the anti-TNF-α-treated patients, no differences can be appreciated in the titers and ND50 variables, indicating that these patients reflected the general pattern of the cohort (**Figure 2**).

Overall, these data show that a high fraction of RMD patients elicit antibodies potentially able to inhibit SARS-CoV-2 infection, most of which target RBD.

### Sustained Neutralizing Antibody Response Over Time Is Maintained in a Fraction of RMD Patients

In order to evaluate the persistence of the antibody response over time and to investigate the cellular immune response to SARS-CoV-2 in RMD patients having resolved the infection, patients were reconvened for a second whole blood sampling in the period August–October 2020 (T2), 3–4 months after T1. For all b-DMARD-treated patients recruited at T2, we had matched samples from T1 (**Supplementary Table 1**). They were predominantly affected by RA (*N* = 18; SpA, *N* = 12). At T2, we also recruited 4 cs-DMARD-treated patients, who were predominantly affected by RA (*N* = 3; SpA, *N* = 1) (**Supplementary Table 1**).

We measured serological responses to SARS-CoV-2 proteins at T2 and compared them to those at T1.

During the intervening time, it was assumed that no cases of re-infection occurred from T1 to T2, as judged by clinical patients monitoring and by the fact that no boost of the anti-RBD and anti-N antibody titers was observed at T2.

**Figure 3:**
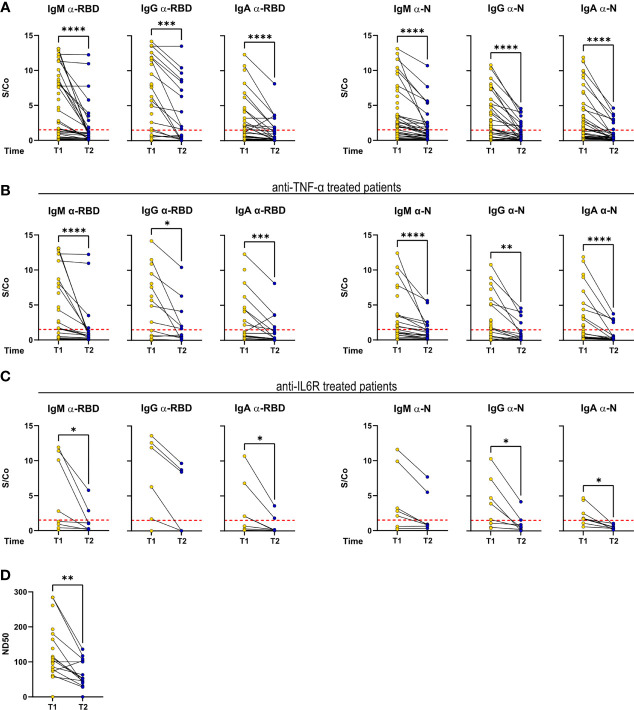
Antibody response is sustained over time in a fraction of RMD patients. IgM, IgG, and IgA levels against RBD and N measured at T1 and T2 in the sera of **(A)** the whole cohort (T1: *N* = 68; T2: *N* = 34), **(B)** α-TNF-α (T1: *N* = 37; T2: *N* = 20), and **(C)** α-IL-6R-treated patients (T1: *N* = 8; T2: *N* = 7). **(D)** Neutralization titers in sera of RMD patients at T1 (*N* = 25) and T2 (*N* = 17). Graphs show individual values, means, and SD. Data from the same patients are linked with a line. Statistical significance was determined using two-tailed Wilcoxon test for paired. *p < 0.05; **p < 0.01; ***p < 0.001; ****p < 0.0001.

Despite an overall decrease in anti-RBD and anti-N titers, as reported (16), a fraction of patients (54.5% of patients found to be serologically positive at T1 and reconvened at T2) maintained IgM, IgG, and IgA titers with sustained or above positivity threshold (**Figure 3A**). Matched T1–T2 titers of patients treated with anti-TNF-α and anti-IL-6R reflected the general pattern of the cohort (**Figures 3B, C**). Concerning the serum neutralization activities, neutralization titers decreased, in agreement with reported data on COVID-19 patients (**Figure 3D**).

### DMARD Treatment Does Not Impact Circulating Monocytes and NK Cells in RMD Patients

To dissect the influence of different DMARDs on the cellular immunological response to SARS-CoV-2 infection, and identify effector populations that are possibly related to infection resolution, we carried out a comprehensive multiparametric flow cytometry analysis of monocytes, NK cells, and B and T lymphocytes from PBMCs of 34 serologically positive RMD patients at T2 (**Supplementary Table 7**). The frequency of classical, intermediate, and non-classical monocytes was comparable in RMD and non-RMD patients (**Supplementary Figure 4A**). When comparing b/ts- and cs-DMARD-treated RMD patients, we could detect a slight reduction of non-classical monocytes in b/ts-DMARD-treated patients (**Supplementary Figure 4A**). Evaluation of the expression of monocyte activation molecules highlighted a trend to increased expression of CD36 in b/ts-DMARD- compared to cs-DMARD-treated patients, while there were no clear differences in the expression of HLA, CCR2, and CD11c (**Supplementary Figure 4B**). Moreover, upon *in vitro* restimulation with poli-IC, CD14^+^ monocytes isolated from b/ts-DMARD-treated patients had increased expression of some pro-inflammatory molecules, especially TNF-α (**Supplementary Figure 4C**). Concerning NK cells and CD3^+^ CD56^+^ cells, we detected a significant increase in the first and a slight decrease in the second in RMD patients as compared to convalescents, with no major impact of cs- or b/ts-DMARD treatment (**Supplementary Figure 4D**).

Overall, these data highlight increased frequencies of NK cells in b/ts-DMARD-treated patients compared to non-RMD individuals. Moreover, monocytes in b/ts-DMARD-RMD-treated patients also showed a limited increase in the expression of activation and effector molecules as compared to cs-DMARDs.

### Anti-TNF-α Treatments Sustain Frequencies of Class-Switched, Memory, and IgG^+^ Memory B Cells

**Figure 4:**
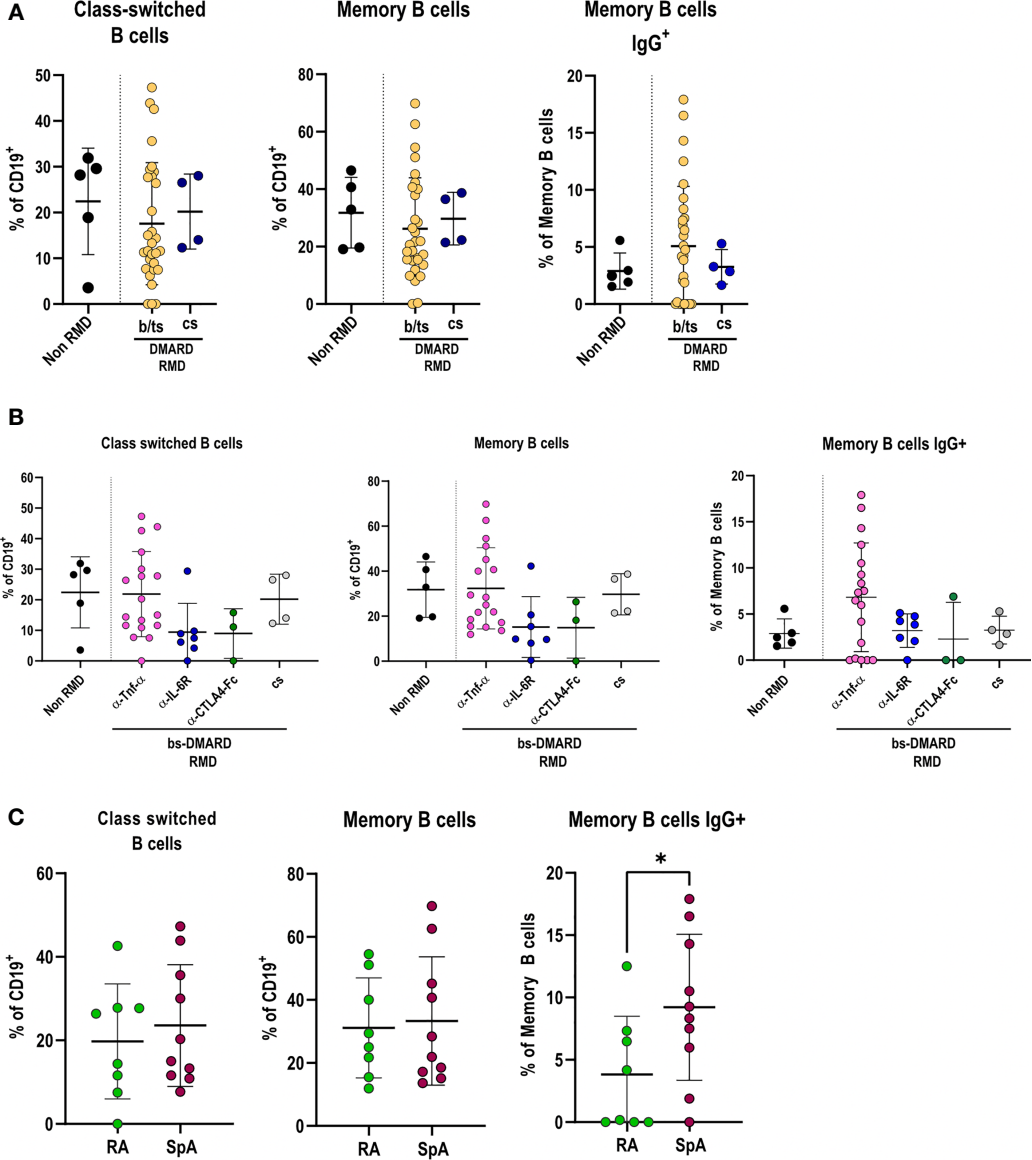
Specific DMARD treatments drive alterations in effector B-cell populations in serologically positive RMD patients. **(A)** Relative frequencies of class-switched, memory, and memory IgG^+^ cells in peripheral blood of non-RMD patients (*N* = 5) and RMD patients treated with b/ts- (*N* = 30) or cs-DMARDs (*N* = 4). **(B)** Relative frequencies of class-switched, memory, and memory IgG^+^ cells in peripheral blood of RMD patients undergoing different b-DMARD (α-TNF-α, *N* = 18; α-IL-6R, *N* = 7; CTLA4-Ig, *N* = 3) or cs-DMARD treatments (*N* = 4), and non-RMD patients (*N* = 5). **(C)** Relative frequencies of class-switched, memory, and memory IgG^+^ cells in α-TNF-α-treated patients sorted according to disease (RA, *N* = 8; SpA, *N* = 10). Graphs show individual values, means, and SD. Statistical significance was determined using two-tailed Mann–Whitney test for unpaired data and Kruskal–Wallis tests to compare unpaired samples between multiple study groups. *p < 0.05.

We proceeded in investigating how DMARD treatments affected the adaptive immune response in RMD patients following SARS-CoV-2 infection. B-lymphocyte frequencies were comparable between RMD patients and non-RMD individuals. However, b/ts-DMARD-treated patients tended to have a lower frequency than cs-DMARD-treated ones (**Supplementary Figure 5A**). When going into the detail of B-lymphocyte subpopulations, we observed that class-switched, memory, and IgG^+^ memory B cells were comparable between RMD patients and non-RMD individuals (**Figure 4A**). Within the b-DMARD groups, patients treated with anti-TNF-α had frequencies of class-switched and memory B cells comparable to non-RMD patients, while the relative abundance of memory IgG^+^ B cells was slightly higher. On the contrary, class-switched and memory B cells had a trend of reduced frequency in patients treated with anti-IL6R and CTLA4-Ig (**Figure 4B**). While frequencies of class-switched and memory B cells were comparable in RA and SpA anti-TNF-α-treated patients, IgG^+^ memory B cells were higher in the last group (**Figure 4C**).

Taken together, these data show that anti-TNF-α treatments in RMD patients sustain frequencies of class-switched, memory, and IgG^+^ memory B cells to levels comparable with non-RMD patients.

### RMD Patients Have Effector T-Cell Frequencies Comparable to Non-RMD Convalescents

**Figure 5:**
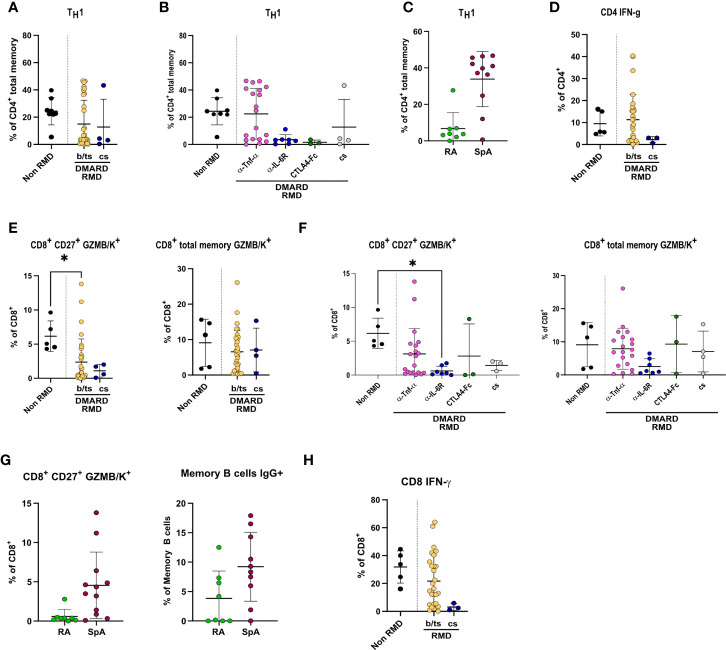
Specific DMARD treatments drive alterations in T_H_1 and effector CD8^+^ T cells in serologically positive RMD patients. Relative frequencies of T_H_1 cells in **(A)** non-RMD patients (*N* = 8) and RMD patients treated with b/ts- (*N* = 31) or cs-DMARDs (*N* = 4). **(B)** RMD patients undergoing different b-DMARD (α-TNF-α, *N* = 19; α-IL-6R, *N* = 7; CTLA4-Ig, *N* = 3) or cs-DMARD treatments (*N* = 4), and non-RMD patients (*N* = 8). **(C)** α-TNF-α-treated patients sorted according to disease (RA, *N* = 8; SpA, *N* = 11). **(D)** Relative frequency of CD4^+^ IFN-γ^+^ in non-RMD patients (*N* = 5) and RMD patients treated with b/ts- (*N* = 28) or cs-DMARDs (*N* = 3). Immunophenotypic analysis of CD8^+^ T lymphocytes by flow cytometry. Relative abundance of CD8^+^ CD27^+^ GZMB/K^+^ and CD8^+^ total memory GZMB/K^+^ subpopulations in PBMC of non-RMD patients (*N* = 5) and RMD patients sorted **(E)** by b/ts- (*N* = 32) and cs-DMARD treatment (*N* = 4) or **(F)** by the individual DMARD treatment (Non-RMD, *N* = 5; α-TNF-α, *N* = 20; α-IL-6R, *N* = 7; CTLA4-Ig, *N* = 3; cs-DMARD, *N* = 3). **(G)** Relative abundance of CD8^+^ CD27^+^ GZMB/K^+^ and CD8^+^ total memory GZMB/K^+^ subpopulations in RA (*N* = 8) and SpA (*N* = 10) treated with α-TNF-α. **(H)** Relative frequency of CD8^+^ IFN-γ^+^ in non-RMD patients (*N* = 5) and RMD patients treated with b/ts- (*N* = 28) or cs-DMARDs (*N* = 3). Graphs show individual values, means, and SD. Statistical significance was determined using two-tailed Mann–Whitney test for unpaired data and Kruskal–Wallis tests to compare unpaired samples between multiple study groups. Statistical significance are reported as : *p < 0.05; **p < 0.01.

CD4^+^ T-cell frequencies were overall significantly lower in RMD patients, especially b-DMARD-treated ones, as compared to convalescents (**Supplementary Figure 5B**). When going into the details of CD4^+^ T-cell populations, we observed that T_H_1 cell frequencies, in spite of being comparable between RMD patients under b- or cs-DMARD treatment and non-RMD convalescents (**Figure 5A**), were higher in anti-TNF-α-treated patients compared to anti-IL6R and CTLA4-Ig treatments (**Figure 5B**). This increased T_H_1 cell frequency in the anti-TNF-α-treated cohort is ascribable to SpA patients, who have significantly higher frequencies as compared to RA patients (**Figure 5C**). We could also observe that cs-DMARD-treated patients had a reduced frequency of CD4^+^ IFN-γ-producing T cells, while b/ts-DMARD-treated patients were comparable to non-RMD ones (**Figure 5D**). Frequencies of CD4^+^ IFN-γ^+^ cells were comparable between anti-TNF-α- and anti-IL6R-treated patients (**Supplementary Figure 6A**).

CD8^+^ T cells were overall comparable in RMD patients as compared to non-RMD convalescents, and did not differ in b- or cs-DMARD-treated ones (**Supplementary Figure 5C**). When dissecting the relative abundance of the different subpopulations, we observed that the abundance of CD8^+^ CD27^+^ GZMB/K^+^ cells, but not CD8^+^ memory GZMB/K^+^, was lower in RMD patients compared to non-RMD individuals, and comparable in b-DMARD- and cs-DMARD-treated patients (**Figure 5E**). Within the b-DMARD-treated cohort, anti-IL6R-treated patients had the lowest level of these effector populations (**Figure 5F**). Within anti-TNF-α-treated patients, SpA patients had increased CD8^+^ CD27^+^ and memory GZMB/K^+^ cells (**Figure 5G**). CD8^+^ IFN-γ-expressing cells were reduced in both b/ts- and cs-DMARD-treated patients as compared to non-RMD, reaching statistical significance only for cs-DMARDs (**Figure 5H**). Frequencies of CD8^+^ IFN-γ^+^ cells were comparable between anti-TNF-α- and anti-IL6R-treated patients (**Supplementary Figure 6B**). Remarkably, although T_REG_ was more abundant in RMD patients compared to non-RMD individuals, we did not detect alterations dictated by DMARD treatment, thus ruling out a role of this population in the modulation of effector B and T cells observed (**Supplementary Figures 6C, D**). The remaining T-cell populations tested in the study (**Supplementary Table 3**) did not show changes among RMD groups.

Overall, these data show that anti-TNF-α-treated patients have increased relative abundance of effector adaptive populations, particularly T_H_1, GZMB/K-expressing CD8^+^ cells, memory, class-switched, and IgG^+^ B cells compared to non-RMD patients.

### Anti-TNF-α-Treated Patients Who Have More Effector Cells Are Generally Asymptomatic

We finally evaluated whether frequencies of these immune cell populations varied according to the COVID-19-related symptomatology experienced by b/ts-DMARD-treated patients (cs-DMARD-treated patients were not evaluated because of the low number). COVID-19 asymptomatic b/ts-DMARD-treated patients had lower frequencies of T_H_1, CD8^+^ CD27^+^ GZMB/K^+^, and memory B IgG^+^ cells (**Supplementary Figures 7A–C**). Within anti-TNF-α-treated patients, COVID-19 asymptomatic subjects had a trend to higher CD8^+^ total memory GZMB/K^+^, memory, and class-switched B cells, while T_H_1 and CD8^+^ CD27^+^ GZMB/K^+^ cells were comparable in symptomatic and asymptomatic subjects (**Supplementary Figures 8A–C**). These data suggest that the increase in adaptive effector populations is more pronounced in COVID-19 asymptomatic RMD patients treated with anti-TNF-α.

## Discussion

The COVID-19 pandemic has shown higher morbidity and mortality rates in fragile patients (1). Immunomodulating agents, such as DMARDs, have been employed in the therapy of COVID-19, in the attempt of halting the inflammatory burst associated with severe disease, and a number of studies have investigated their possible influence on infection incidence and severity (9, 22–27).

In the clinical practice, b/ts-DMARD or cs-DMARDs represent usual treatments for RMD patients, and there is concern whether their protracted use could cause a higher risk of developing a biased immune response upon SARS-CoV-2 infection due to the underlying immune system dysfunction. However, the incidence of COVID-19 in the RMD population was reported to be comparable to the general population (9, 27), and seroprevalence to SARS-CoV-2 was similar between RMD patients and the general population after at least 1 month from infection (19, 28).

In this context, a thorough investigation of the impact of DMARDs on the immune response to SARS-CoV-2 infection in RMD patients would significantly improve the management of RMD patients during the pandemic. To address this unmet need, we analyzed the humoral and cellular immune responses in patients affected by RA or SpA, who experienced COVID-19 in spring 2020 with moderate symptoms or without symptoms.

We observed that COVID-19 symptomatic RMD patients had a higher seropositivity rate than the asymptomatic ones, in line with other studies in normal individuals showing that anti-SARS-CoV-2 antibody correlates with disease severity (29, 30), and DMARDs did not alter a successful seroconversion rate (20). However, the magnitude of the antibody response to SARS-CoV-2 RBD and N proteins was moderately reduced in RMD patients compared to non-RMD. Noteworthy, the antibody neutralizing ability was comparable between RMD and non-RMD patients, indicating that DMARDs did not hamper antibody maturation and production of antibodies potentially able to block viral infection. Neutralizing antibodies are the result of an antibody maturation process consisting of accumulated somatic mutations over months in convalescent individuals, generally associated with prolonged exposure to the antigen (31, 32). The evidence that efficient antibody maturation is maintained during DMARD treatments gives a reassuring message for RMD patients. However, when dissecting the influence of individual b-DMARDs, we observed that CTLA4-Ig (Abatacept) significantly reduces antibody levels against viral proteins, posing a warning on this specific treatment that deserves further investigation.

Concerning the cellular immune response, we did not observe marked differences in the B- and T-cell populations between RMD patients treated with b/ts-DMARD or cs-DMARDs, compared to non-RMD individuals. Importantly, our study does not present data about SARS-CoV-2-specific cell populations, but alterations following SARS-CoV-2 infection. Noteworthy, RMD patients treated with CTLA4-Ig and anti-IL-6R showed lowest relative frequencies of class-switched, memory, and memory IgG^+^ B and T_H_1 cells. On the other side, RMD patients treated with anti-TNF-α drugs have higher relative abundance of effector adaptive populations, particularly T_H_1, GZMB/K-expressing CD8^+^ cells, memory, class-switched, and IgG^+^ B cells comparable to non-RMD patients. Finally, COVID-19 asymptomatic subjects treated with anti-TNF-α had a trend to higher effector CD8^+^, memory, and class-switched B cells than the symptomatic ones, while T_H_1 cells were comparable in the two groups. Other studies reported that ongoing therapy with CTLA4-Ig (Abatacept) and anti-IL-6R was not associated with a worse clinical course of COVID-19 (33). IL-6 inhibition was reported to be effective in reducing mortality of subgroups of patients with markedly high C-reactive protein concentrations and with low lactate dehydrogenase concentrations (34). On the contrary, treatment with anti-TNF-α has been associated with a lower hospitalization odds ratio as compared to other b- or cs-DMARDs (35). Our data improve the knowledge in the field, suggesting that patients treated with anti-TNFα, but not CTLA4-Ig, could have a more efficient effector immune response upon re-exposure. Indeed, it is known that CTLA4-Ig, through binding of CD80/86 on antigen-presenting cells, inhibits T-cell activation and memory B-cell formation. A study reported that RA patients that were HBV occult carriers experienced viral reactivation upon CTLA4-Ig treatment (36). In addition, CTLA4-Ig treatment, in association with traditional DMARDs, significantly reduces the humoral response to the pandemic 2009 influenza A/H1N1 vaccine (33, 37) and to the pneumococcal conjugate vaccine in RA patients (38). Our observation prompts additional trials aimed at confirming whether CTLA4-Ig is contraindicated in the treatment of RMD patients with COVID-19, being detrimental to the elicitation of the immune response to vaccination. In addition, it would be important to compare results with RMD patients treated with anti-CD20 antibody in a dedicated study, since this cohort was under-represented in our study.

Anti-TNFα drugs neutralize a major component of the cytokine response that is part of the damaging excess inflammatory phase of COVID-19. Our data suggest that anti-TNF-α treatments may be beneficial to COVID-19 outcome through elicitation of protective immune response in RMD patients. In addition, our data highlight a differential immune response to COVID-19 between RA and SpA patients, the former showing a lower propensity than SpA patients to induce effector T- and B-lymphocyte populations. RA patients could be potentially more susceptible to SARS-CoV-2 re-infection, requiring an *ad hoc* vaccination regimen to boost a long-lasting protective immunity (39).

Over the pandemic course, we observed the emergence of variants of concern (VoC) that may cause evasion from the protective immunity conferred by previous infection or vaccination (40). Our data regarding the presence of neutralizing antibodies suggest that RMD patients under DMARD treatment who previously experienced COVID-19 should be able to mount an immune response against VoCs. Moreover, studies on T cells reported that the impact of mutations present in VoCs is limited and that the majority of CD4+ and CD8+ T-cell responses are preserved in both vaccinated and natural infection conditions being directed against conserved T epitopes (41–44). Therefore, we expect that effector immune cell populations should contribute to the protection from re-infection with VoCs as well. However, as SARS-CoV-2 antibodies and T effector cells are lower in patients treated with anti-IL-6R and CTLA4-Fc, these treatments might cause increased susceptibility to re-infection with VoCs.

Our data contribute to the field providing a whole picture of the humoral and cellular immune responses in RMD patients, reassuring the use of DMARD treatments during COVID-19. Among b-DMARDs, TNF-α inhibitors elicit functional antibodies to SARS-CoV-2 and adaptive effector populations available to counteract possible re-infections.

The primary strategy worldwide to contain the COVID-19 pandemic is vaccination. Based on our data, there is no reason to assume that DMARD treatment could impair vaccination efficacy in RMD patients, as we detect both neutralizing antibodies and effector immune cell populations in our cohort. However, the lower abundance of effector immune cells in patients treated with anti-IL-6R and CTLA4-Fc might suggest an attenuated response in these patients.

## Limitations of the Study

We envision the following limitations in our study.

Due to the limited availability of the PCR test during the recruitment period (May–June 2020), we assessed COVID-19 only through serological analyses. To minimize loss of detection of the seropositive patients, we used a low cutoff to score serological positivity. Nevertheless, we cannot exclude an underestimation of the seropositive patients, possibly affecting the frequency of symptomatic and asymptomatic patients’ comparison. Moreover, our study did not appropriately investigate patients whose serological response was possibly impaired by the DMARD treatment itself, such as those undergoing anti-CD20 treatments, who deserve dedicated studies. In addition, this study was conducted in a real-life setting, and reflected the relative sizes of the treatment schedules in place at ASST Gaetano Pini-CTO Institute. Consequently, the small sample size for some DMARD treatment groups might limit the conclusions regarding such treatments. Finally, our study does not present data about SARS-CoV-2-specific immune cell populations, but alterations following SARS-CoV-2 infection.

## Data Availability Statement

The raw data supporting the conclusion of this article will be made available by the authors, upon request.

## Ethics Statement

Recruitments occurred at the ASST Gaetano Pini-CTO Institute in Milan (Italy) and were under ethical approval by the Ethics Committee Milano Area 2 (MAINSTREAM protocol: approval number 407; END-COVID: approval number 331). All patients signed informed consent. The patients/participants provided their written informed consent to participate in this study.

## Author Contributions

Project conceptualization and study design were performed by RG, SA, RC, and EF. RG designed the experiments, interpreted the results, and wrote the paper. RC, EF, EM, MBi, AGor, and AB recruited patients, collected clinical samples, and contributed to manuscript writing. AGob, MBo, AF, and EZ performed statistical analyses. EZ contributed to data interpretation and manuscript writing. AF, PG, VB, MS, MM, and TF processed samples and performed experiments. MBo, EP, LD, and EM performed serological analyses. RF, AF, and PG performed immunophenotyping experiments. MC contributed to flow cytometry analysis. SN contributed to data interpretation. All authors contributed to the article and approved the submitted version.

## Funding

This research was supported by the projects COiMMUNITY (ID 1842163) to RG and MAINSTREAM (ID 1835032) to SA, both funded by Regione Lombardia and co-funded under POR FESR 2014-2020 resources; by the project COVID-2020-12371640 to SA funded by Italian Ministry of Health; and by an unrestricted grant from Fondazione “Romeo ed Enrica Invernizzi”; by Fondazione Cariplo (INNATE-CoV) to LM.

## Conflict of Interest

Author EM is employed by Dia.Pro (Diagnostic Bioprobes srl, Milan, Italy).

The remaining authors declare that the research was conducted in the absence of any commercial or financial relationships that could be construed as a potential conflict of interest.

## Publisher’s Note

All claims expressed in this article are solely those of the authors and do not necessarily represent those of their affiliated organizations, or those of the publisher, the editors and the reviewers. Any product that may be evaluated in this article, or claim that may be made by its manufacturer, is not guaranteed or endorsed by the publisher.
