# Supplementary material for: Immunosuppressant Treatment in Rheumatic Musculoskeletal Diseases Does Not Inhibit Elicitation of Humoral Response to SARS-CoV-2 Infection and Preserves Effector Immune Cell Populations
Source: Front Immunol. 2022 Jun 10;13:873195. doi: 10.3389/fimmu.2022.873195 (PMC9226581; doi:10.3389/fimmu.2022.873195)
Supplement: Supplementary file 1 [file DataSheet_1.pdf]

frontiers

Supplementary Material

Supplementary Figures

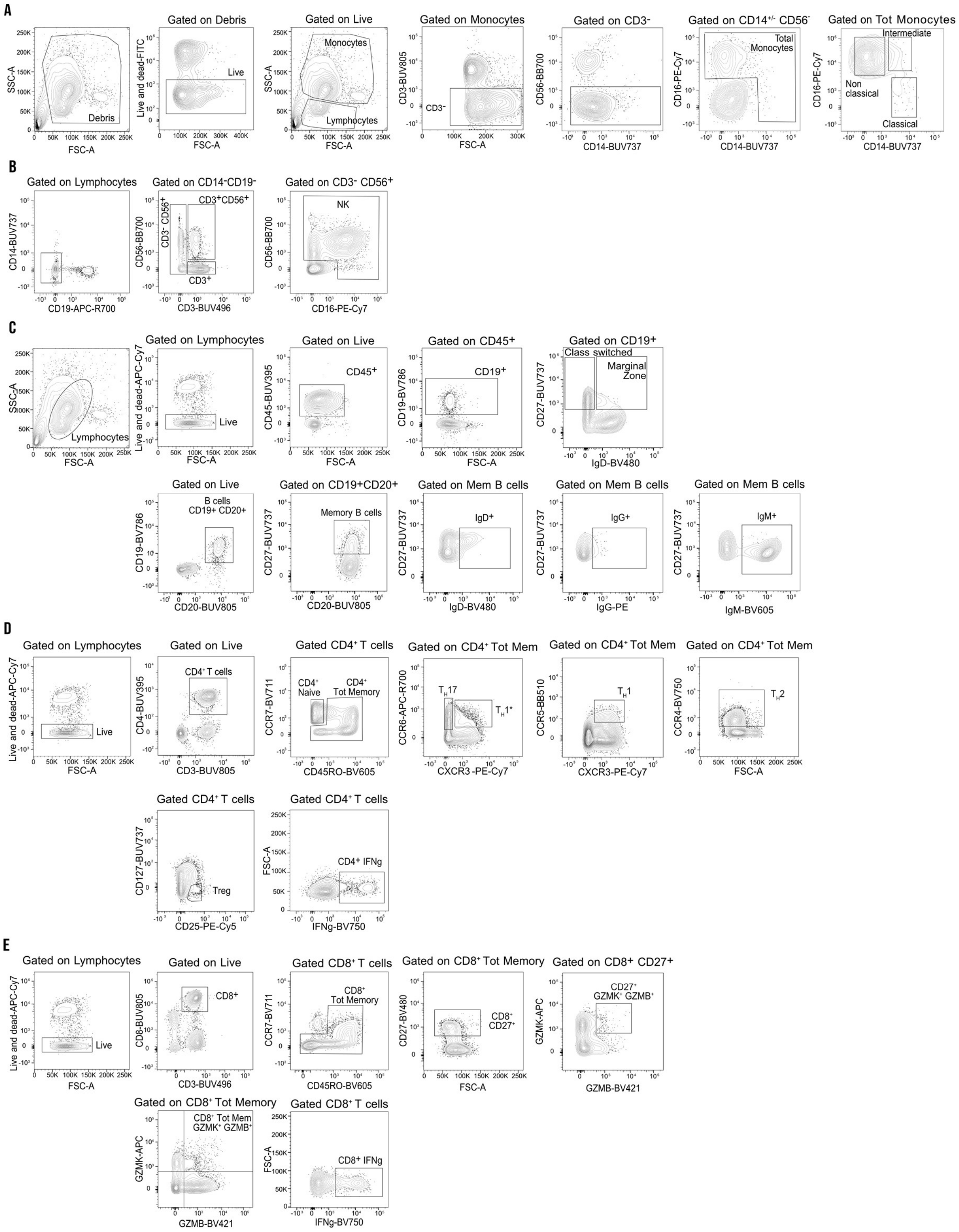

Supplementary Figure 1. Gating strategies for flow cytometry analyses. Gating strategies used for the identification of: **(A)** classical, intermediate and non-classical monocytes; **(B)** NK cells and $CD3^+$ $CD56^+$ cells; **(C)** B cell subpopulations (in particular: class-switched, memory and memory IgG^+); **(D)** T helper cell subpopulations (in particular: T_H1, $CD4^+$ IFN-γ^+ and T_{REG}); **(E)** $CD8^+$ T cell subpopulations (in particular: $CD8^+$ $CD27^+$ GZMB/K^+, $CD8^+$ Total Memory GZMB/K^+ and $CD8^+$ IFN-γ^+).

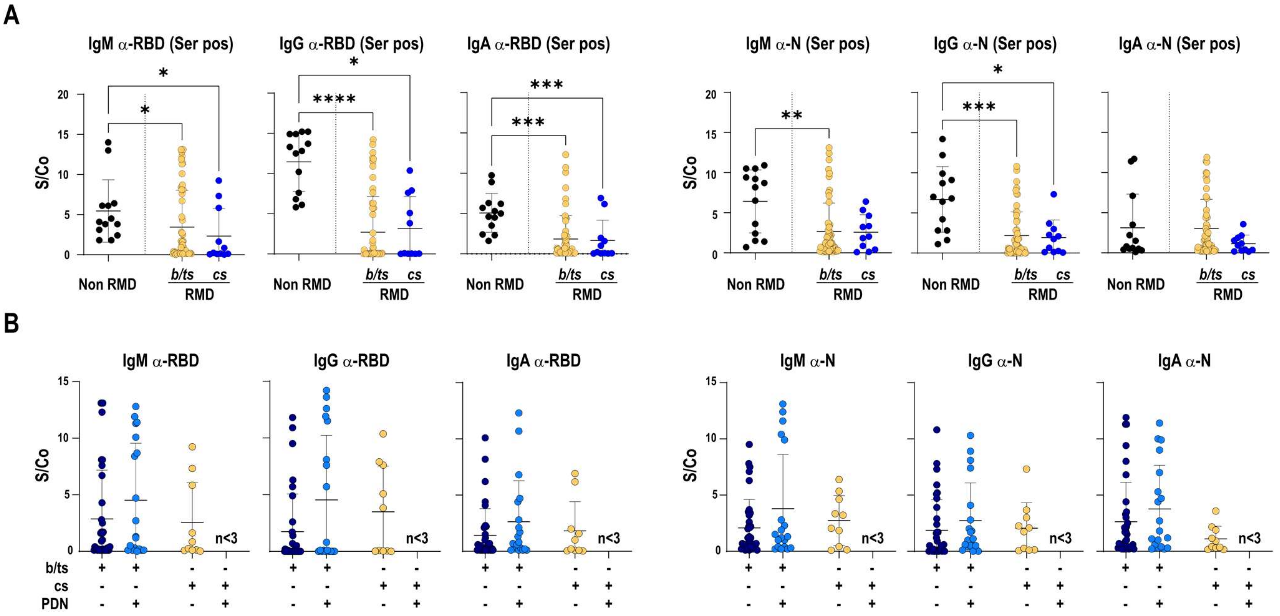

Supplementary Figure 2. Magnitude of the anti-RBD and anti-N antibody response in RMD patients. (A) IgM, IgG and IgA levels against RBD and N measured in the sera of non RMD patients not under immunosuppressant treatment (N=13) and RMD patients undergoing b/ts- (N=55) or cs-DMARD (N=11) treatment. **(B)** IgM, IgG and IgA levels against RBD and N measured in the sera of RMD patients undergoing b/ts- or cs-DMARD treatment alone or coupled with prednisone (PDN) (b/ts-DMARD N=36, b/ts-DMARD+PDN N=19, cs-DMARD N=10). Graphs show individual values, means and SD. Statistical significance was determined using Kruskal-Wallis tests to compare unpaired samples between multiple study groups. * p<0.05; ** p<0.01; *** p<0.001; **** p<0.0001.

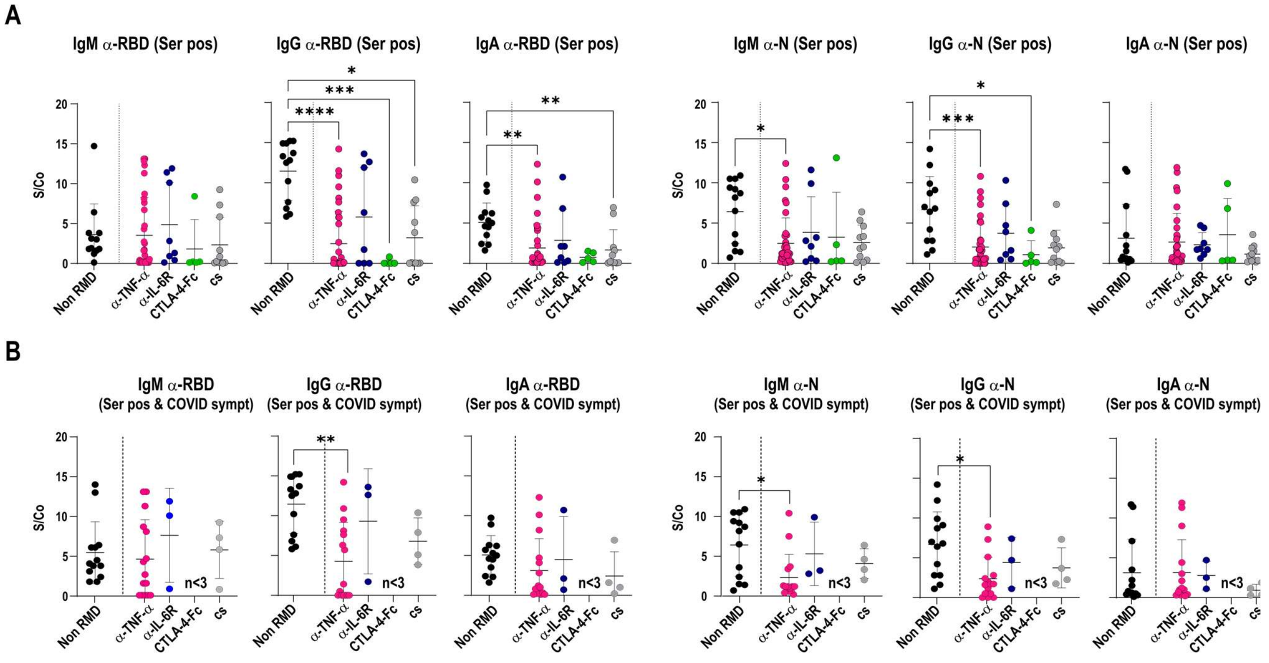

Supplementary Figure 3. Magnitude of the anti-RBD and anti-N antibody response in RMD patients, undergoing different DMARD treatments, as compared to non RMD individuals. IgM, IgG and IgA levels against RBD and N measured in the sera of **(A)** serologically positive (anti-TNF-α N=37, anti-IL-6R N=8, CTLA4-Ig N=5, cs-DMARD N=11) and **(B)** serologically positive and COVID-19 symptomatic RMD patients undergoing different b-DMARD (anti-TNF-α N=15, anti-IL-6R N=3) or cs-DMARD (N=4) treatments, and non RMD patients (N=13). Graphs show individual values, means and SD. Statistical significance was determined using Kruskal-Wallis tests to compare unpaired samples between multiple study groups. * $p<0.05$; ** $p<0.01$; *** $p<0.001$; **** $p<0.0001$.

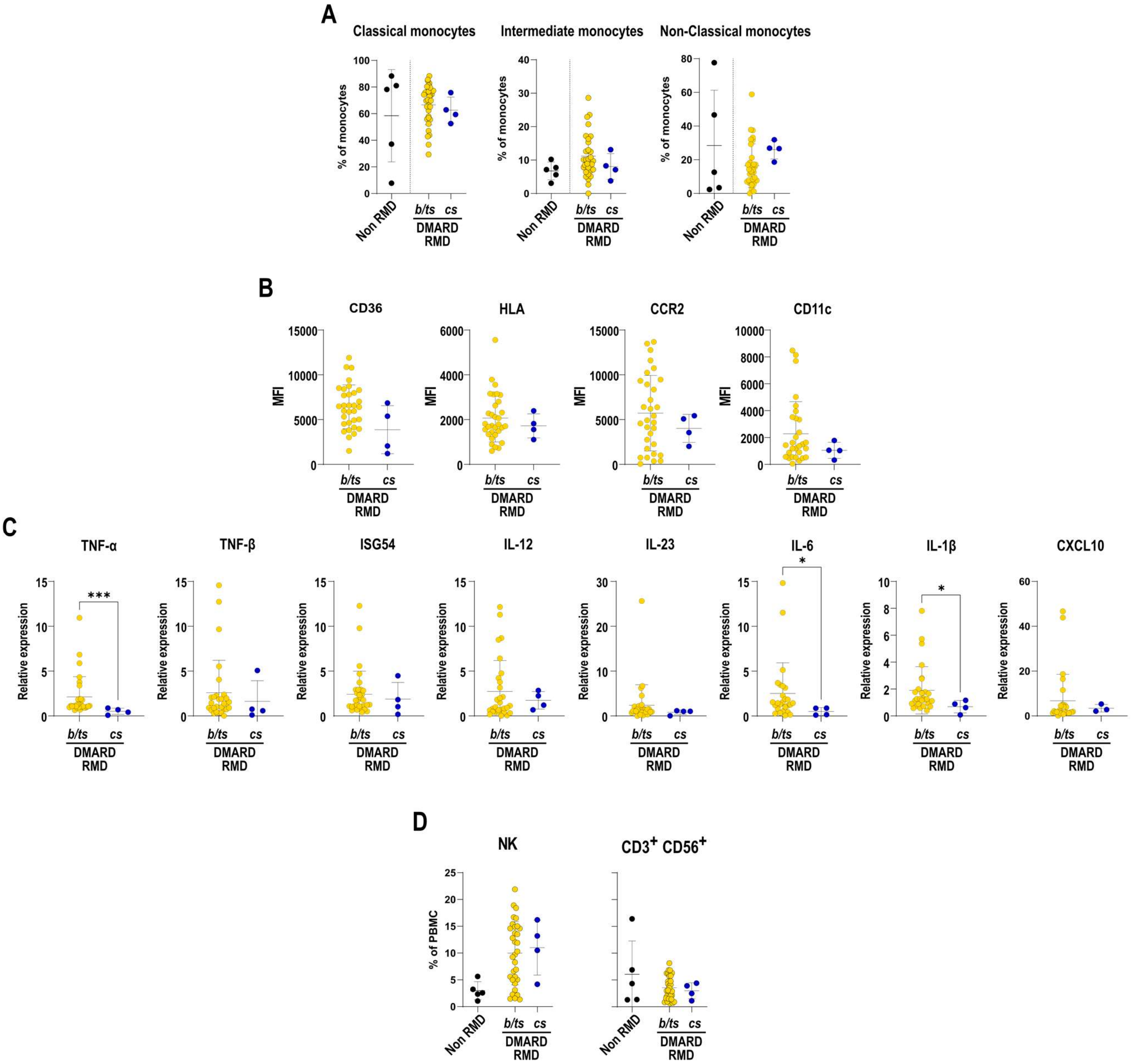

Supplementary Figure 4. DMARD treatment has a minor impact on the innate immune response following SARS-CoV-2 infection in serologically positive RMD patients. (A) Relative frequencies of classical, intermediate and non-classical monocytes in peripheral blood of non RMD patients (N=5) and RMD patients treated with b/ts- (N=32) or cs-DMARDs (N=4). **(B)** Mean fluorescence intensity (MFI) of CD36, HLA, CCR2 and CD11c on $CD14^+$ monocytes in peripheral blood of RMD patients treated with b/ts- (N=32) or cs-DMARDs (N=4). **(C)** $CD14^+$ monocytes were isolated from PBMCs of RMD patients treated with b/ts- (N=30) or cs-DMARDs (N=4) and restimulated *in vitro* with poli-IC. RT-qPCR showing the relative expression of TNF-α, TNF-β, ISG54, IL-12, IL-23, IL-6, IL-1β, CXCL10. **(D)** Relative frequencies of NK and $CD3^+$ $CD56^+$ cells in peripheral blood of non RMD patients (N=5) and RMD patients treated with b/ts- (N=32) or cs-DMARDs (N=4). Graphs show individual values, means and SD. Statistical significance was determined using two-tailed–Mann-Whitney test to pairwise groups comparison. Multiple Kruskal-Wallis test was used to compare unpaired samples between study groups. * p<0.05, *** p<0.001.

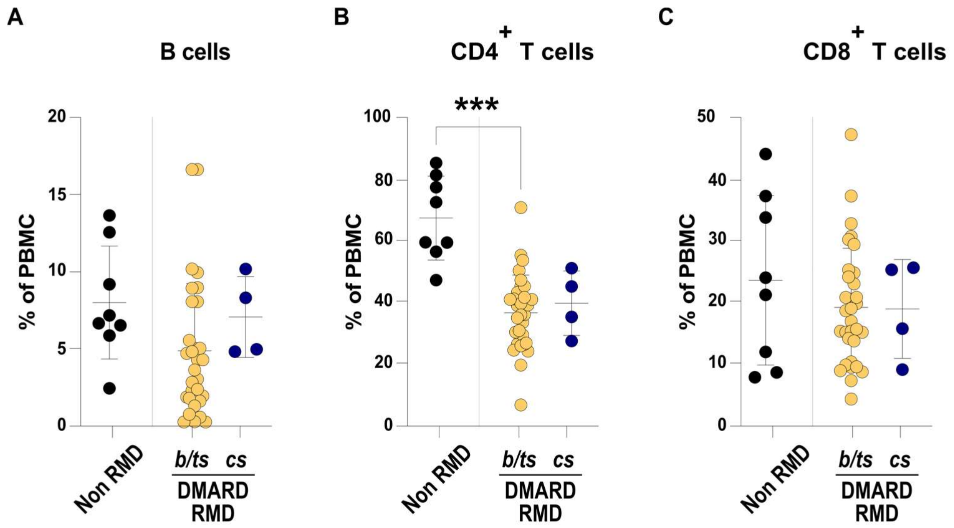

Supplementary Figure 5. Immune signatures of B and T lymphocyte populations in serologically positive RMD patients with COVID-19 collected after recovery from the infection. PBMCs from RMD patients treated with b/ts-DMARD (N=30) or with cs-DMARD (N=4) and non RMD patients (N=8), were analyzed by multiparametric flow cytometry. The frequencies of **(A)** B lymphocytes, **(B)** $CD4^+$ and **(C)** $CD8^+$ T lymphocytes were analyzed in relation to treatments. Graphs show individual values, means and SD. Multiple Kruskal-Wallis test was used to compare unpaired samples between study groups. *** $p<0.001$.

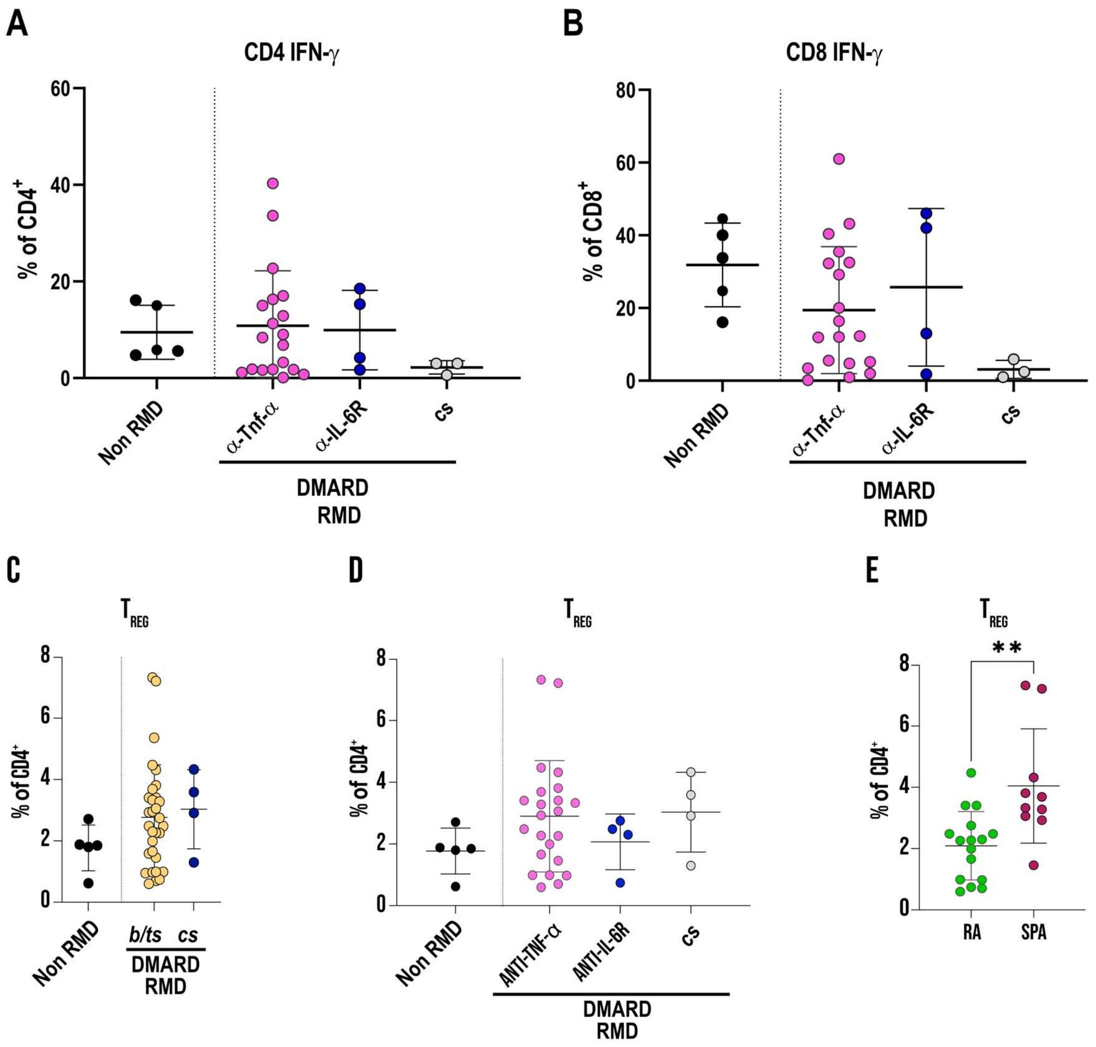

Supplementary Figure 6. Relative frequencies of **(A)** CD4+ IFN-γ+ and **(B)** CD8+ IFN-γ+ in RMD patients undergoing different b-DMARD (anti-TNF-α N=19, anti-IL-6R N=4) or cs-DMARD treatments (N=3), and non RMD patients (N=5). TREG frequencies in peripheral blood of: **(C)** non RMD patients (N=5) and RMD patients treated with b/ts- (N=31) or cs-DMARDs (N=4); **(D)** RMD patients undergoing different b-DMARD (anti-TNF-α N=23, anti-IL-6R N=4) or cs-DMARD treatments (N=4), and non RMD patients (N=5); **(E)** anti-TNF-α-treated patients sorted according to disease (RA N=16; SpA N=10). Graphs show individual values, means and SD. Multiple Kruskal-Wallis test was used to compare unpaired samples between study. ** $p<0.01$.

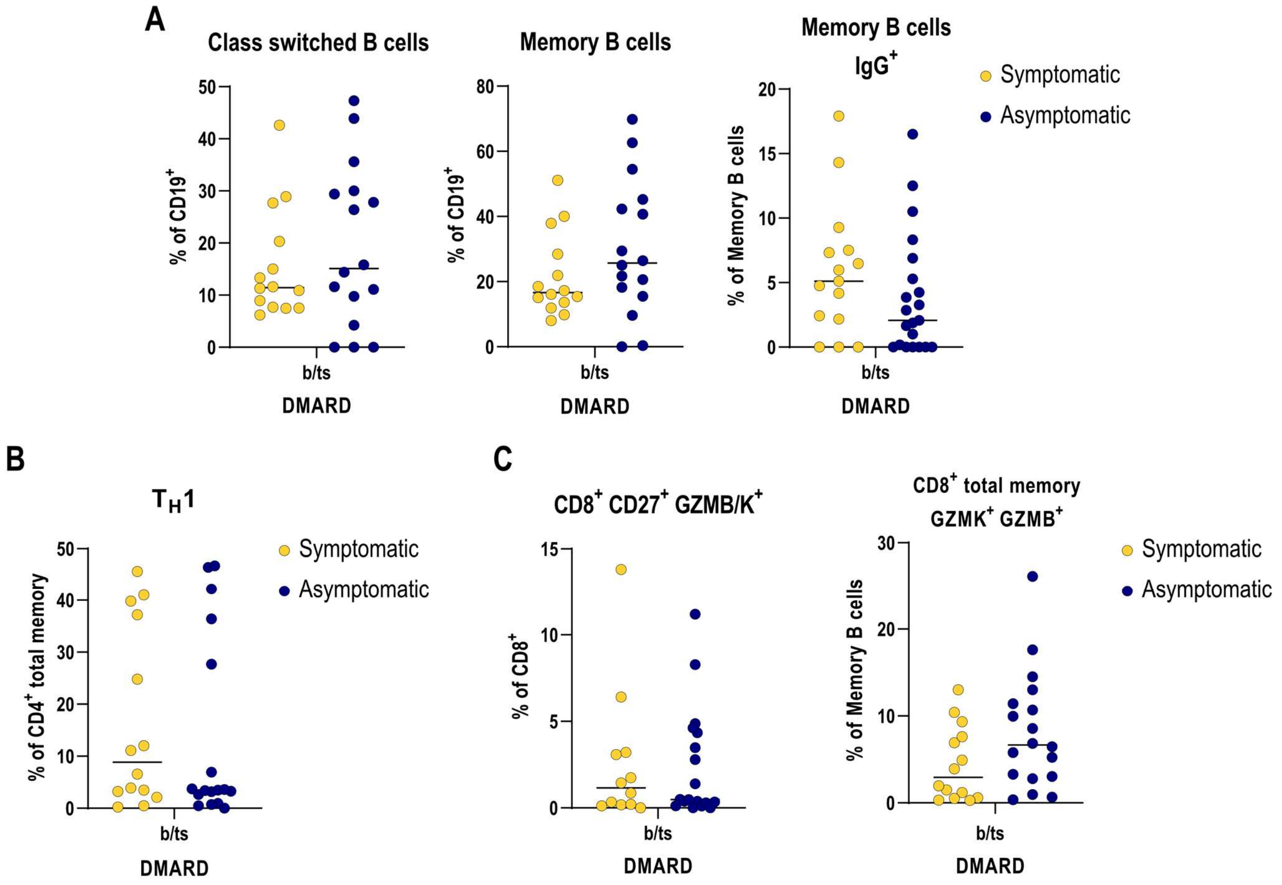

Supplementary Figure 7. Relative frequency of **(A)** class-switched, memory and memory IgG^+ cells, **(B)** T_H1 and **(C)** of $CD8^+$ $CD27^+$ $GZMB/K^+$ and $CD8^+$ total memory $GZMB/K^+$ in b/ts-DMARD-treated patients sorted according to COVID-19 related symptomatology (Symptomatic N=14; asymptomatic N=17). Graphs show individual values and means. Statistical analysis was performed by two-tailed Mann-Whitney test.

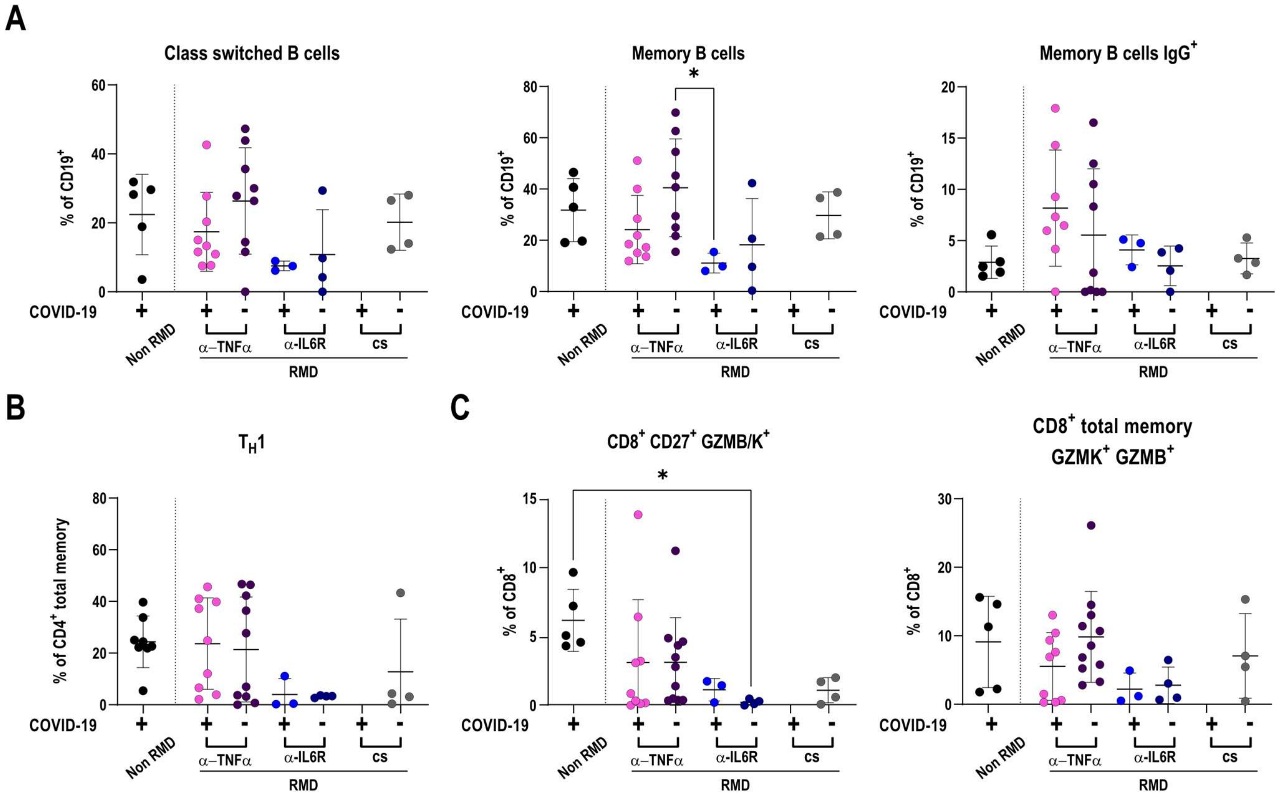

Supplementary Figure 8. Relative frequency of **(A)** class-switched, memory and memory IgG^{+} cells, **(B)** T$_{H}$1 and **(C)** of CD8^{+} CD27^{+} GZMB/K^{+} and CD8^{+} total memory GZMB/K^{+} in non RMD patients (N=5) and RMD patients treated with anti-TNF-α, anti-IL-6R and cs-DMARDs, sorted according to COVID-19 related symptomatology (symptomatic: COVID-19 +; asymptomatic: COVID-19 -) (anti-TNF-α COVID-19 + N=9; anti-TNF-α COVID-19 – N=9; anti-IL-6R COVID-19 + N=3; anti-IL-6R COVID-19 – N=4; cs-DMARD COVID-19 – N=4). Graphs show individual values, means and SD. Multiple Kruskal-Wallis test was used to compare unpaired samples between study groups. * $p<0.05$.

Supplementary Tables

Supplementary Table 1. Clinical and demographic characteristics of the study population.
RA rheumatoid arthritis, SpA spondyloarthritis, HCQ hydroxychloroquine, csDMARDs conventional synthetic disease-modifying anti-rheumatic drugs, b/tsDMARDs biological/targeted synthetic disease-modifying anti-rheumatic drugs, TNF tumor necrosis factor, IL interleukin. JAKi Janus kinase inhibitors, MTX methotrexate, HCQ Hydroxychloroquine, PDE4i phosphodiesterase 4 inhibitor.

	T1			T2			
	**Total**	**RA**	**SpA**	**Total**	**RA**	**SpA**	**Non RMD**
**Number**	**358**	**200**	**158**	**36**	**23**	**13**	**13**
**Age (y); mean (SD)**	52.8 (13.6)	52.8 (13.4)	52.7 (13.6)	54.8 (13.8)	57.3 (15.3)	50.3 (9.6)	45.6 (18.8)
**Female, n (%)**	230 (64.2)	155 (77.5)	75 (47.5)	25 (69.4)	19 (82.6)	6 (46.2)	7 (53.8)
**Anti-rheumatic treatment**							
**b-DMARDs (%)**	277 (77.4)	141 (70.5)	136 (86.1)	30 (83.3)	18 (78.2)	12 (92.3)	
**anti-TNF-α**	173	61	112	20	8	12	
**CTLA4-Ig**	42	39	3	3	3	0	
**Anti-IL6R**	35	34	1	7	7	0	
**Anti-IL17**	14	0	14	0	0	0	
**Anti-CD20**	6	6	0	0	0	0	
**Anti-IL1**	3	1	2	0	0	0	
**Anti-IL22/23**	4	0	4	0	0	0	
**ts-DMARDs (%)**	22 (6.1)	16 (8)	6 (3.8)	2 (5.6)	2 (8.7)	0 (0)	
**JAKi**	17	16	1	2	2	0	
**PDE4i**	5	0	5	0	0	0	
**cs-DMARDs mono (%)**	59 (16.5)	43 (21.5)	16 (10.1)	4 (11.1)	3 (13.0)	1 (7.7)	
**MTX**	44	32	12	4	3	1	
**Leflunomide**	1	0	1	0	0	0	
**HCQ**	12	11	1	0	0	0	
**Sulfasalazine**	2	0	2	0	0	0	
**cs-DMARDs association - MTX (%)**	112 (31.3)	75 (37.5)	37 (23.4)	10 (27.8)	6 (26.1)	4 (30.8)	
**Prednisolone (%)**	105 (29.3)	80 (40)	25 (15.8)	14 (38.9)	12 (52.2)	2 (15.4)	

Supplementary Table 2. Characterization of COVID-19-related symptomatology.

	**All RMD patients N (%) 358**	**RMD seropositive patients N (%) 66**
**Asymptomatics**	281 (78.5%)	41 (62.1%)
**Cough**	47 (13.1%)	16 (24,2%)
**Asthenia**	46 (12.8%)	13 (19.7%)
**Fever (< 37.5°C)**	24 (6.7%)	7 (10.6%)
**Fever (> 37.5°C)**	40 (11.2%)	13 (19.7%)
**Myalgia**	37 (10.3%)	9 (13.6%)
**Pharyngodynia**	31 (8.6%)	10 (15.1%)
**Dyspnea**	16 (4.4%)	5 (7.5%)
**Smell loss**	17 (4.7%)	11 (16.6%)
**Taste loss**	17 (4.7%)	10 (15.1%)
**Nausea or vomit**	8 (2.2%)	2 (3.0 %)
**Diarrhea**	16 (4.5%)	5 (7.5%)
**Hospitalized**	5 (1,4%)	4 (6.0 %)
**ICU**	0	0

Supplementary Table 3. Markers used for the identification of specific immune cells by flow cytometry.

Subpopulation of immune cells analyzed in the study	Marker staining used for flow cytometry
**CD4 T CELLS**	
**T_H1**	$CCR7^+$ $CD45RO^{+/-}$ $CXCR3^+$ $CCR5^+$
**T_H1***	$CD4^+$ $CCR7^+$ $CD45RO^{+/-}$ $CXCR3^+$ $CCR6^+$
**T_H17**	$CD4^+$ $CCR7^+$ $CD45RO^{+/-}$ $CXCR3^-$ $CCR6^+$
**Treg**	$CD4^+$ $CD127^{low}$ $CD25^+$
**T_H2**	$CD4^+$ $CCR7^+$ $CD45RO^{+/-}$ $CCR4^+$
**CD4 NAÏVE**	$CD4^+$ $CCR7^+$ $CD45RO^-$
**CD4 TOTAL MEMORY**	$CD4^+$ $CCR7^+$ $CD45RO^{+/-}$
**$CD4^+$ IFN**	$CD4^+$ IFN-γ^+
**CD8 T CELLS**	
**CD8 NAÏVE**	$CD8^+$ $CCR7^+$ $CD45RO^-$
**CD8 TOTAL MEMORY**	$CD8^+$ $CCR7^+$ $CD45RO^{+/-}$
**$CD8^+$ $CD27^+$ GZMBK**	$CD8^+$ $CD27^+$ GZMB/K^+
**$CD8^+$ TOTAL MEMORY GZMBK**	$CD8^+$ $CCR7^+$ $CD45RO^{+/-}$ $GZMB^+$ $GZMK^+$
**$CD8^+$ IFNγ**	$CD8^+$ IFN-γ^+
**B cells**	
**B cells**	$CD14^-$ $CD19^+$
**CLASS-SWICHED B CELLS**	$CD45^+$ $CD19^+$ $CD27^+$ IgD^-
**MEMORY B CELLS**	$CD45^+$ $CD19^+$ $CD27^+$ $CD20^+$
**MARGINAL ZONE**	$CD19^+$ $CD27^+$ IgD^+
**memory IgD**	$CD45^+$ $CD19^+$ $CD27^+$ $CD20^+$ IgD
**memory IgM**	$CD45^+$ $CD19^+$ $CD27^+$ $CD20^+$ IgM
**memory IgG**	$CD45^+$ $CD19^+$ $CD27^+$ $CD20^+$ IgG
**Macropopulations**	
**Classical Monocytes**	$CD3^-$ $CD14^-$ $CD56^{+/-}$ $CD16^-$ $CD14^+$
**Intermediate Monocytes**	$CD3^-$ $CD14^-$ $CD56^{+/-}$ $CD16^+$ $CD14^+$
**Non-classical Monocytes**	$CD3^-$ $CD14^-$ $CD56^{+/-}$ $CD16^+$ $CD14^-$
**NK**	$CD3^-$ $CD56^+$ $CD16^{+/-}$
**$CD3^+$ $CD56^+$**	$CD3^+$ $CD56^+$

Supplementary Table 4. Antibodies used for flow cytometry.

Antibody target (eventual alias in brackets)	Fluorocrome	Clone	Titration x FACS	Company	Catalog #
**CD194 (CCR4)**	BV750	161	1:100	BD	746980
**CD 21b**	BUV661	MNC2	1:100	BD Prototype	624285
**CD121a**	BUV615	HIL1R-M1	1:100	BD Prototype	624297
**CD127**	BB700	HIL-7R-M21	1:100	BD	566398
**CD127**	BUV737	HIL-7R-M21	1:200	BD	612794
**CD137 (41BB)**	BUV786	4B4-1 LIGAND	1:100	BD	741000
**CD137 (41BB)**	APC	4B4-1 LIGAND	1:100	BD	561702
**CD138**	PECF594	MI15	1:100	BD	9123873
**CD14**	BUV737	M5E2	1:100	BD	612763
**CD161**	FITC	191B8	1:100	MILTENYI BIOTEC	130-113-592
**CD16**	PECY7	3G8	1:100	BIOLEGEND	302016
**CD183 (CXCR3)**	BUV395	1C6/CXCR3	1:100	BD	565223
**CD183 (CXCR3)**	PECY7	1C6/CXCR3	1:100	BD	560831
**CD185 (CXCR5)**	PE	2G8	1:100	BD	551959
**CD186 (CXCR6)**	BV480	13B1E5	1:100	BD	746431
**CD19**	APCR700	BIH19	1:100	BD	564977
**CD19**	BV786	SJ25C1	1:100	BD	563325
**CD195 (CCR5)**	FITC	HEK/1/85a	1:100	BIOLEGEND	313705
**CD196 (CCR6)**	APCR700	11A9	1:100	BD	565173
**CD197 (CCR7)**	BV711	150503	1:100	BD	566602
**CD198 (CCR8)**	BV786	433H	1:100	BD	747574
**CD357 (GITR)**	BV421	V27-580	1:100	BD	566423
**CD20**	BUV805	2H7	1:100	BD	564917
**CD22**	BV750	HIB22	1:100	BD	747421
**CD24**	BV711	ML5	1:100	BD	563401
**CD25**	PECY5	M-A251	1:200	BD	555433
**CD27 BV480**	BV480	L128	1:100	BD	566139
**CD27**	BV786	L128	1:100	BD	563327
**CD27**	BUV737	L128	1:100	BD	564301
**CD39**	BV711	TU66	1:100	BD	563680
**CD223 (LAG3)**	BV480	T47-530	1:100	BD	746609
**CD244**	PECF594	2-69	1:200	BD	564881
**CD273 (PD-L2)**	APCR700	HIH18	1:100	BD	565189
**CD274 (PD-L1)**	BV650	MIH1	1:100	BD	563740
**CD278 (ICOS)**	PECY7	C398.4A	1:100	BIOLEGEND	313520
**CD278 (ICOS)**	BV421	DX29	1:100	BD	562901
**CD279 (PD1)**	BV750	EH12.1	1:200	BD	747446
**CD294 (CRTH2)**	PECF594	BM16	1:100	BD	563501
**CD3**	BUV496	UCHT1	1:100	BD	564809

**CD3**	BUV 805	UCHT1	1:200	BD	612895
**CD357 (GITR)**	BV421	V27-580	1:100	BD	566423
**CD366 (TIM3)**	BB515	7D3	1:100	BD	565568
**CD38**	BB700	HIT2	1:100	BD	566445
**CD4**	BUV737	SK3	1:100	BD	612748
**CD4**	BUV 395	SK3	1:100	BD	563550
**CD45**	BUV395	HI30	1:100	BD	563791
**CD45RO**	BV605	UCHL1	1:100	BD	562791
**CD56**	BB700	NCAM 16.2	1:100	BD	566573
**CD8**	BUV 805	RPA-T8	1:200	BD	749366
**CD152 (CTLA4)**	PECY5	BNI3	1:50	BD	555854
**CD8**	BUV 563	RPA-T8	1:200	BD	565695
**FOXP3**	PECF594	259D/C7	1:100	BD	562421
**GRANZ B**	BV421	GB 11	1:100	BD	563389
**GRANZ K**	Alexa Fluor647	G3H69	1:100	BD	566655
**GNLY PE**	PE	DH2	1:50	BIOLEGEND	348004
**CD272 (BTLA)**	PE	MIH26	1:50	BIOLEGEND	344506
**HLADR**	BUV661	G46-6	1:100	BD	612980
**HLDR.DP.DQ**	BV750	TU39	1:200	BD	746979
**TIGIT**	BV605	741182	1:100	BD	747841
**IFN-γ**	BV750	B27	1:100	BD	566357
**IgD**	BV480	IA6-2	1:50	BD	566187
**IgG**	PE	G18-145	1:50	BD	555787
**IgA**	FITC	M24A	1:50	MERKMILLIPORE	CBL114F
**IgM**	BV605	G20-127	1:50	BD	562977
**IL10**	PECF594	JES3-19F1	1:100	BD	562400
**IL17**	FITC	ebio64DEC17	1:50	INVITROGEN	11-7179-42
**IL4**	PECY7	MP4-25D2	1:50	BIOLEGEND	500824
**IL6**	BV421	MQ2-13A5	1:100	BD	563279
**IL21**	Alexa Fluor647	3A3-N2.1	1:100	BD	560493
**IL13**	PE	JES10-5A2	1:100	BD	554571
**IL2**	BV510	5344-111	1:100	BD	563265
**PD1**	BUV563	MIH1	1:100	BD	741423
**TNF-α**	BUV395	MAb11	1:100	BD	563996
**gm-csf**	BB 630 P2	BrD2-21C11	1:200	BD	614294
**TNF-β**	FITC	LTX-21	1:200	INVITROGEN	BMS105FI
**IL16**	PE	14,1	1:100	BIOLEGEND	519106
**LIVE AND DEAD**	FIX VIAB 780		1:2000	BD	565388

Supplementary Table 5. Classification criteria of symptoms.

Major symptoms	Minor symptoms
Fever >37.5°C	Fever <37.5°C
Dyspnea	Pharyngodynia
Cough	Myalgia
Ageusia	Asthenia
Anosmia	Nausea or vomit
	Diarrhea

Supplementary Table 6. Frequency of COVID-19 symptomatic patients stratified by disease and therapy.

	**Total**	**RA**	**SpA**
**Number (% of total)**	**77 (21.5)**	**39 (19.5)**	**38 (24.1)**
**b/ts-DMARD (% of symptomatic)**	46 (59.8)	17 (43.6)	29 (76.3)
**cs-DMARDs mono (% of symptomatic)**	7 (9.1)	6 (15.4)	1 (2.6)
**cs-DMARDs association - MTX (% of symptomatic)**	24 (31.2)	16 (41)	8 (21.1)

Supplementary Table 7. Frequencies of immune cells analysed by flow cytometry and described in the study.

		NON RMD	DMARD RMD		Single treatments				Only anti-TNF-α	
**IMMUNE CELLS (% of parental cells)**	**parental cells**	**no treatment**	**b/ts**	**cs**	**CTLA4-Ig**	**anti-TNF-α**	**anti-IL-6R**	**cs**	**RA**	**SpA**
**CLASS-SWICHED B CELLS/CD19$^+$**	CD19$^+$	22,44	17,56	20,20	8,97	21,87	9,43	20,20	19,75	23,56
**MEMORY B CELLS/**		31,78	26,23	29,73	14,87	32,34	15,17	29,73	31,14	33,30
**MEMORY B CELLS IgG**	Memory B cells	2,89	5,07	3,27	2,30	6,82	3,21	3,27	4,90	9,79
**Th1**	CD4$^+$ total memory	24,38	14,94	12,75	1,63	22,47	3,53	12,75	6,76	33,89
**CD4$^+$ IFNγ**	CD4$^+$	9,46	11,31	2,21	20,78	10,83	9,93	2,21	4,21	14,69
**CD8$^+$ CD27$^+$ GZMBK**	CD8$^+$	6,15	2,37	1,10	2,80	3,11	0,61	1,43	0,59	4,55
**CD8$^+$ TOTAL MEMORY GZMBK**		9,10	6,55	7,07	9,31	7,91	2,53	7,07	4,13	10,43
**CD8$^+$ IFNγ**		31,84	21,71	3,11	33,50	19,40	25,70	3,11	13,77	22,68
**Classical Monocytes**	Monocytes	58,47	66,66	62,65						
**Intermediate Monocytes**		6,76	11,18	8,07						
**Non-classical Monocyted**		28,52	16,53	26,00						
**NK**	PBMC	2,97	9,98	11,02						
**CD3$^+$ CD56$^+$**		6,05	3,55	2,98						
**B cells**		7,82	4,65	6,87						
**CD4$^+$**		67,59	36,44	39,63						
**CD8$^+$**		23,49	19,07	18,81						
